# Evaluating the abiotic synthesis potential and the stability of building blocks of life beneath an impact-induced steam atmosphere

**DOI:** 10.3389/fmicb.2023.1032073

**Published:** 2023-04-03

**Authors:** Zongbin Zhang, Haofan Jiang, Pengcheng Ju, Lu Pan, Joti Rouillard, Gentao Zhou, Fang Huang, Jihua Hao

**Affiliations:** ^1^Deep Space Exploration Laboratory, University of Science and Technology of China, Hefei, China; ^2^CAS Key Laboratory of Crust-Mantle Materials and Environments, School of Earth and Space Sciences, University of Science and Technology of China, Hefei, China; ^3^State Key Laboratory of Continental Dynamics, Northwest University, Xi’an, China; ^4^Shaanxi Key Laboratory of Early Life and Environment, Department of Geology, Northwest University, Xi’an, China; ^5^Centre for Star and Planet Formation, Globe Institute, University of Copenhagen, Copenhagen, Denmark; ^6^CAS Center for Excellence in Comparative Planetology, University of Science and Technology of China (USTC), Hefei, Anhui, China

**Keywords:** origin of life, primitive Earth, impact events, abiotic synthesis, steam atmosphere, building blocks of life

## Abstract

A prerequisite for prebiotic chemistry is the accumulation of critical building blocks of life. Some studies argue that more frequent impact events on the primitive Earth could have induced a more reducing steam atmosphere and thus favor widespread and more efficient synthesis of life building blocks. However, elevated temperature is also proposed to threaten the stability of organics and whether life building blocks could accumulate to appreciable levels in the reducing yet hot surface seawater beneath the steam atmosphere is still poorly examined. Here, we used a thermodynamic tool to examine the synthesis affinity of various life building blocks using inorganic gasses as reactants at elevated temperatures and corresponding steam pressures relevant with the steam-seawater interface. Our calculations show that although the synthesis affinity of all life building blocks decreases when temperature increases, many organics, including methane, methanol, and carboxylic acids, have positive synthesis affinity over a wide range of temperatures, implying that these species were favorable to form (>10^–6^ molal) in the surface seawater. However, cyanide and formaldehyde have overall negative affinities, suggesting that these critical compounds would tend to undergo hydrolysis in the surface seawaters. Most of the 18 investigated amino acids have positive affinities at temperature <220°C and their synthesis affinity increases under more alkaline conditions. Sugars, ribose, and nucleobases have overall negative synthesis affinities at the investigated range of temperatures. Synthesis affinities are shown to be sensitive to the hydrogen fugacity. Higher hydrogen fugacity (in equilibrium with FQI or IW) favors the synthesis and accumulation of nearly all the investigated compounds, except for HCN and its derivate products. In summary, our results suggest that reducing conditions induced by primitive impacts could indeed favor the synthesis/accumulation of some life building blocks, but some critical species, particularly HCN and nucleosides, were still unfavorable to accumulate to appreciable levels. Our results can provide helpful guidance for future efforts to search for or understand the stability of biomolecules on other planets like Mars and icy moons. We advocate examining craters formed by more reducing impactors to look for the preservation of prebiotic materials.

## 1. Introduction

How life originated on the Earth (or beyond) has been a long-lasting puzzle for decades (Haldane, 1929; Oparin and Morgulis, 1938). The origin of life relies on several environmental factors, including the availability of various molecular building blocks, such as amino acids, sugars, and nucleotides. Apart from this, chemical emergence of life is also believed to require high levels of key reactive compounds, including cyanide (HCN and CN^–^), urea [CO(NH_2_)_2_], and formaldehyde (HCHO). Cyanide is thought to be one essential base to form nucleobases and other N-bearing biomolecules in the primitive ocean (Sanchez et al., 1967; Yadav et al., 2020; Pérez-Fernández et al., 2022), thus constituting a critical starting point for the “RNA World” hypothesis; urea is suggested to be a useful reagent for prebiotic phosphorylation reactions (Lohrmann and Orgel, 1971; Powner et al., 2009), whereas formaldehyde – a major hydrolysis product of cyanide – is believed to be a critical starting material for the synthesis of sugars on primitive Earth (Orgel, 1998).

There are two major sources commonly put forward for the molecules aforementioned on the primitive Earth: endogenous synthesis and extraterrestrial delivery (Chyba and Sagan, 1992). The former mainly includes synthesis processes in various high-energy environments, including the hydrothermal alteration of ultramafic rocks (e.g., McCollom and Seewald, 2007; Cardace et al., 2015; McDermott et al., 2015; Ménez et al., 2018), the heating or radiation of the primitive atmosphere and surface waters (Stribling and Miller, 1987; Kobayashi et al., 1998; Parkos et al., 2018; Li et al., 2022; Zang et al., 2022), and the lightning [e.g., the widely known Urey-Miller experiment; (Miller, 1953)]. The latter source, extraterrestrial delivery, is primarily *via* the impact of extraterrestrial materials (meteorites, comets, and interplanetary dust particles) on the early Earth, which was much more frequent than today (Ryder, 2002). These extraterrestrial materials, particularly carbonaceous chondrites and comets, can contain substantial levels of various organic compounds (Kvenvolden et al., 1970; Cronin and Chang, 1993; Furukawa et al., 2019). Additionally, people suggested that shock-induced synthesis of various organics molecules could happen during large impact events (Bar-Nun et al., 1970; Furukawa et al., 2009; Goldman et al., 2010; Martins et al., 2013; Sugahara and Mimura, 2015; Takeuchi et al., 2020).

Although life relies on liquid water, polymerization reactions that lead to functional biomolecules (e.g., polynucleotides, polypeptides) are largely dehydration processes. That is one major challenge for the origin of life studies as one usually needs very high reactant concentrations for chemical evolution to happen in simulative experiments–for example, molar level of HCN used in synthesis reactions (Orgel, 1998; Yadav et al., 2020) vs. the <10^–6^ molar concentrations that have been estimated for the primitive ocean (Miyakawa et al., 2002). Earlier simulation studies had assumed a reducing primitive atmosphere where synthesis and accumulation of organic compounds would be more favored (Miller, 1953). For example, photochemical synthesis of HCN is shown to have appreciable yields in a relatively reducing atmosphere rich in CH_4_, C_2_H_2_, or CO (Schlesinger and Miller, 1983; Rimmer and Rugheimer, 2019). However, chemical analyses of ancient zircons implied that redox state of the early mantle was similar to the modern (Trail et al., 2011) and thus the early volcanic gas should be less reducing or weakly oxic, similar to the modern. Photochemical models further suggested that hydrogen escape to the space would only allow a weakly to moderately reducing atmosphere on the early Earth, with a partial pressure of H_2,*g*_ inferior to 10^–2^ bar (Kuramoto et al., 2013; Hao et al., 2019). These results contradict the proposed view of highly reducing atmosphere enabling pervasive abiotic synthesis on the primitive Earth.

Recently, several studies pointed out that a reducing atmosphere could rather be periodically induced by impacts on the primitive Earth (Hashimoto et al., 2007; Genda et al., 2017a,b; Schaefer and Fegley, 2017; Parkos et al., 2018; Zahnle et al., 2020). In the proposed scenario, reducing impactors would tend to equilibrate with the induced steam atmosphere and release high levels of reducing gasses, such as H_2_, CH_4_, and NH_3_ (Zahnle et al., 2020; Pearce et al., 2022). It was further estimated that photochemistry in this reducing atmosphere would generate high levels of reactive species critical to prebiotic chemistry, such as HCN (Benner et al., 2020; Pearce et al., 2022). However, the impact events led to not only a more reducing atmosphere but also a hot surface environment. For instance, impact simulations suggested that a large impactor (corresponding from 100 to 250 km in diameter) would generate a steam atmosphere (>100°C) lasting for 1 to 100 years (Segura et al., 2002; Zahnle et al., 2020; Pearce et al., 2022). Under these circumstances, surface waters presumably in equilibrium with the steam atmosphere would also be hot. Whether or not photochemically synthesized life building blocks could accumulate to high concentrations in the reducing yet hot surface waters has not been carefully examined in the previous models.

Generally, increasing temperature would imply a much faster hydrolysis rate of the building blocks of life. For example, under neutral pH conditions, the half-life time of HCN decreases from several years at ambient temperatures to mere hours at 100°C (Miyakawa et al., 2002). However, the net reaction of synthesis and hydrolysis processes would be governed by the thermodynamic driving force. Indeed, previous studies already suggested that some small organics could reach thermodynamic equilibrium with geologically abundant gaseous species under elevated temperatures (Shock, 1988, 1990; Lazcano and Miller, 1994; Franiatte et al., 2008). In this study, we applied thermodynamic calculations to assess the chemical affinities for synthesis/degradation of the building blocks of life under the simulative post-impact conditions. We focused on the surface seawater where photochemically synthesized organics and the relevant reactive compounds from the atmosphere would precipitate and dissolve. Thus, the surface seawater might steadily reach equilibrium with the impact-induced reducing and hot atmosphere.

The organics studied here were selected so as to represent the main groups of biomolecules (amino acids, sugars, nucleobases, and nucleosides) and also include some of the major organics found in meteorites, comets, and interplanetary particles (Hayes, 1967; Pizzarello et al., 2006; Goesmann et al., 2015; Aponte et al., 2020; Glavin et al., 2020; Oba et al., 2022) (summarized in Supplementary Table 1). We have also considered some critical compounds used in prebiotic synthesis [cyanide (Yadav et al., 2020), formaldehyde, and urea], as well as common metabolic materials [methanol (Russell and Nitschke, 2017), glycolic acid, and pyruvic acid]. Methane is also considered here since it was thought to be a critical greenhouse gas keeping the early Earth warm under the faint young Sun (Feulner, 2012).

## 2. Materials and methods

### 2.1. Post-impact surface conditions

As mentioned above, large impact events could induce a reducing steam atmosphere lasting for years or longer. Thus, in this study, we focused on simulating post-impact surface waters beneath the steam atmosphere (Table 1). Specifically, we chose to model a wide range of temperatures (100–340°C, allowing a steam atmosphere above the hot surface seawater) and, correspondingly, the atmospheric pressure as the saturation pressure of water vapor. The atmospheric composition (except for H_2_O) was adopted from Zahnle et al. (2020); the H_2_O vapor pressure is assumed to be in equilibrium with liquid water. The surface water’s redox state was assumed to be dominated by the diffusion of H_2,*g*_ from the atmosphere, i.e., by H_2,*g*_ partial pressure and solubility in the water. The surface seawater pH would be largely controlled by equilibration with the atmospheric CO_2_ (and other acidic gasses, such as HCl) as well as alteration of the impactor (Kadoya et al., 2020). The partial pressure of acidic gases and the extent of the water-rock interaction, which are key parameters to estimate the pH, are poorly constrained and might evolve in time and space. Given these uncertainties, we decided to study a wide range of pH (2–12) in the models, thus encompassing various potential scenarios.

**TABLE 1:** Model settings for the impact calculations.

Environmental parameter	Settings	Consideration and references
Temperature	100–340°C	Temperature allowing the condensation of steam atmosphere to liquid water ocean
Total pressure (predominantly as H_2_O,_g_)	Saturation pressure of water	A global water ocean in equilibrium with steam atmosphere
pCO_2,g_	1 bar	Zahnle et al., 2020
pCO,_g_	10^–3^ bar	Zahnle et al., 2020
pH_2,g_	10^0.9^ bar	Zahnle et al., 2020
pNH_3,g_	10^–2.5^ bar	Zahnle et al., 2020
pN_2,g_	1 bar	Zahnle et al., 2020
pH	2–12	A wide range representing different hydrothermal settings (Gulmann et al., 2015; Seewald et al., 2015)

### 2.2. Abiotic synthesis reaction affinities

Gibbs free energy (*G*_*r*_) of a reaction:


(1) a⁢A+b⁢B⇌c⁢C+d⁢D

is defined as


(2) $$△\,G_{r}=△\,G_{r}^{{}^{\circ}}+2.303\,R\,T\,l\,o\,g_{10}\,Q,$$

where *R* is ideal gas constant (8.314 J/(mole⋅K), *T* is temperature in Kelvin (=273.15+ °C), and *Q* is reaction quotient $\frac{{\left\{A\right\}}^{a}\times {\left\{B\right\}}^{b}}{{\left\{C\right\}}^{c}\times {\left\{D\right\}}^{d}}$ ({*X*} refers to the activity/fugacity of species X). When the reaction reaches equilibrium, it turns into a special case:


(3) $$△\,G_{r}^{{}^{\circ}}=-2.303\,R\,T\,l\,o\,g_{10}\,K,$$

where *K* is the reaction constant at a given temperature and pressure. The reaction affinity is defined as:


(4) $$A_{r}=-2.303\,R\,T\,l\,o\,g_{10}\,\frac{Q}{K}.$$

The actual value of *A*_*r*_ represents the maximum amount of energy released (*A*_*r*_ > 0) or required (*A*_*r*_ < 0) for the reaction to reach thermodynamic equilibrium (*A*_*r*_ = 0). Therefore, if *Q* > *K*, *A*_*r*_ is negative and the overall reaction tends to move backward; if *Q* < *K*, *A*_*r*_ is positive and the overall reaction tends to move forward; if *Q* = *K*, *A*_*r*_ is zero and the reaction is at equilibrium.

Here, we calculated the reaction affinities of abiotic synthesis reactions (Supplementary Table 2). We first calculated the reaction quotient by using the partial pressures of gaseous bases from Zahnle et al. (2020) and assuming the activity of the product as 10^–6^. The selection of the product activity is arbitrary here for better comparison between different organics and also reflects the fact that chemical evolution reactions usually requires appreciable levels (>10^–6^ molal) of dissolved bases. Reaction constants for the abiotic synthesis reactions under both ambient and hydrothermal conditions were calculated using the Deep Earth Water (DEW) model [(Sverjensky et al., 2014); free access online],^1^ which was built on the revised Helgeson-Kirkham-Flowers (HKF) equation of state for aqueous species (Shock et al., 1997). We then used the calculated affinity to evaluate whether the synthesis/stability of 10^–6^ molal product is thermodynamically favorable (positive affinity) or not (negative affinity) in hot surface seawater after impact events. For protonated organics (e.g., HCOOH and HCOO^–^), we firstly calculated the pKa(s) of the protonation reaction(s) and then used Henderson-Hasselbalch Equation to calculate speciation for a given pH (Supplementary Figure 1). The reported affinity for protonated species will be the sum of percentage of different protonated species multiplied by their corresponding affinities, e.g., $A_{t\,o\,t\,a\,l}=A_{H\,C\,O\,O\,H}^{*}\,\frac{\left[H\,C\,O\,O\,H\right]}{\left[H\,C\,O\,O\,H\right]+\left[H\,C\,O\,O^{-}\right]}+A_{H\,C\,O\,O^{-}}^{*}$ $\frac{\left[H\,C\,O\,O^{-}\right]}{\left[H\,C\,O\,O\,H\right]+\left[H\,C\,O\,O^{-}\right]}$. Its Internally consistent thermodynamic properties (*G*, *H*, *S*, *C*_*p*_, and HKF parameters) of the organics come from a compilation of previous studies (Shock and Helgeson, 1990; Shock et al., 1992; Schulte and Shock, 1993; Plyasunov and Shock, 2001) and are summarized here in Supplementary Table 3.

It is notable that we considered separately two C sources in all synthesis reactions: CO and CO_2_. In the primitive atmosphere, CO could come from volcanic outgassing and photo-dissociation of CO_2_; the latter might reach photochemical equilibrium in a steady state (Kasting, 2014). However, such gaseous equilibrium does not translate to the thermodynamic equilibrium of their aqueous species in surface waters due to the kinetic barrier of CO_2_ reduction under ambient conditions. A similar example is the co-existence of H_2_ and O_2_ in modern and early atmospheres (Kasting, 1993), which certainly did not reach thermodynamic equilibrium with liquid H_2_O under ambient conditions (Hao et al., 2019). Here, we also calculated the thermodynamic equilibrium constant for the reaction: *CO*_2,*g*_ + *H*_2,*g*_*CO*_*g*_ + *H*_2_*O* and compared with the reaction quotient calculated by the reference composition in Table 1 (Supplementary Figure 2). The comparison clearly shows that CO_2_ and CO are not in aqueous equilibrium, and there is a reduction tendency of CO_2_ to generate CO by H_2_. Therefore, using CO and CO_2_ separately as C source in abiotic synthesis reactions does not necessarily result in same affinity values, and in this study, the synthesis affinity value with CO would be generally lower than CO_2_ due to the addition of positive affinity for the above-mentioned reaction.

## 3. Results

### 3.1. C1 species

**FIGURE 1:**
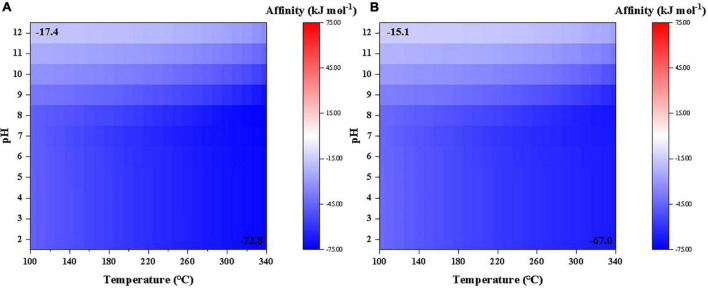
Synthesis affinity of cyanide (HCN,_aq_ + CN^–^) using **(A)** CO,_g_ and **(B)** CO_2,g_ as the carbon source.

According to our calculations, the affinity of cyanide synthesis to reach 10^–6^ molal level from either CO or CO_2_ as carbon source is overall negative under a wide range of temperature and pH conditions investigated in this study (Figure 1). Synthesis affinity using CO as carbon base (Figure 1A) is generally lower than using CO_2_ (Figure 1B). It is notable that the affinity decreases with increasing temperatures but increases at elevated pHs.

**FIGURE 2:**
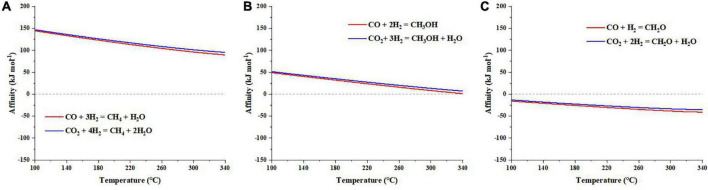
Synthesis affinity of **(A)** methane (CH_4,aq_), **(B)** methanol (CH_3_OH,_aq_), and **(C)** formaldehyde (HCHO,_aq_) using CO,_g_ (in red) and CO_2,g_ (in blue) as the carbon source. Given that these species are not protonated/deprotonated at the range of pH investigated here, pH has no effects on the results.

**FIGURE 3:**
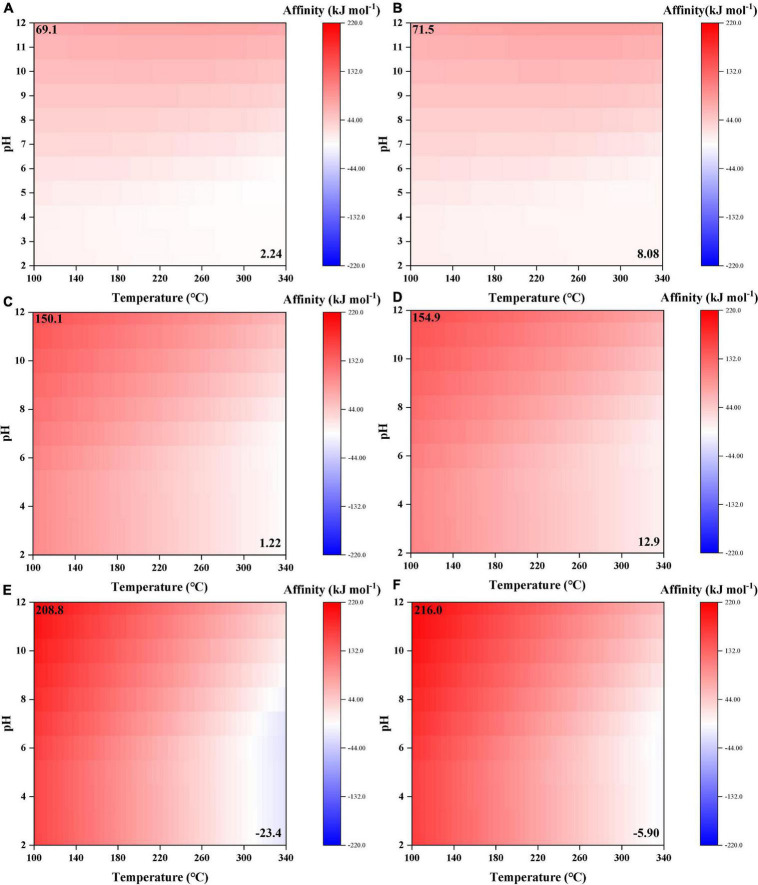
Synthesis affinity of formic [HCOOH,_aq_ + HCOO^–^; **(A,B)**], acetic [CH_3_COOH,_aq_ + CH_3_COO^–^; **(C,D)**], and propanoic [C_2_H_5_COOH,_aq_ + C_2_H_5_COO^–^; **(E,F)**] acids using **(A,C,E)** CO,_g_ and **(B,D,F)** CO_2,g_ as the carbon source.

Unlike cyanide, our calculations suggest that synthesis affinities of methane and methanol are positive under the explored temperature conditions (Figures 2A, B). However, the synthesis affinity of formaldehyde is overall negative (Figure 2C), like cyanide. Comparing the three C1 organics, there is a steadily decreasing trend of synthesis affinities along with increasing oxidation state of C, i.e., synthesis affinity: CH_4_ > CH_3_OH > HCHO. However, the synthesis affinity of formic acid + formate, which has the most oxidized C, is overall positive (Figures 3A, B), inconsistent with the observed trend. Like cyanide, synthesis affinity of C1 organics decreases at high temperatures but increases at elevated pHs (for formic acid). Moreover, abiotic synthesis of these C1 organics using CO as the carbon source has an overall lower affinity than using CO_2_.

### 3.2. C2–C3 species

Similar to formic acid, acetic acid and propanoic acid have positive synthesis affinities (to reach 10^–6^ molal) at almost the whole range of temperature and pH (Figures 3C–F). Moreover, synthesis affinities are overall lower using CO (Figures 3C, E) as the carbon source than CO_2_ (Figures 3D, F). Furthermore, the elevation of pH results in increasing reaction affinity. However, comparing the absolute affinity values of these carboxylic acids, the elongation of alkane chain seems to result in increasing affinities at relatively low temperatures (< 260°C) but decreasing affinities at high temperatures (>260°C). At T > 300°C and pH < 8, the synthesis affinity of propanoic acid even turns negative.

**FIGURE 4:**
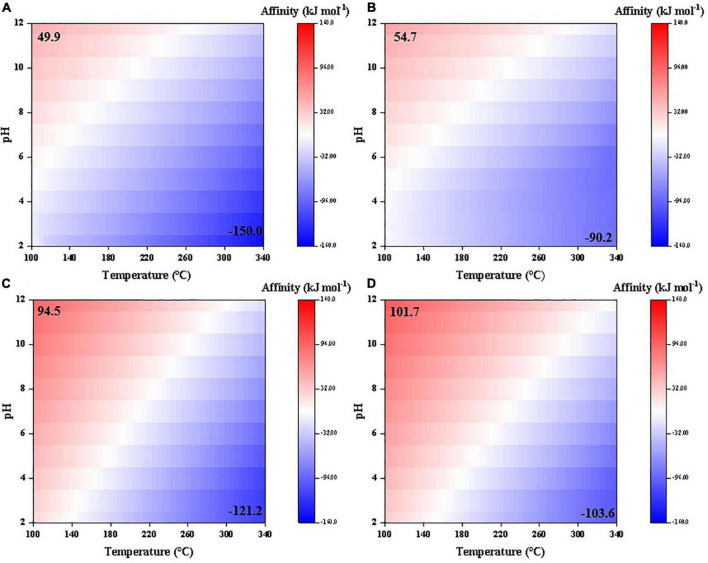
Synthesis affinity of glycolic acid [CH_2_OHCOOH,_aq_ + CH_2_OHCOO^–^; **(A,B)**] and pyruvic acid [CH_3_COCOOH,_aq_ + CH_3_COCOO^–^; **(C,D)**] using **(A,C)** CO,_g_ and **(B,D)** CO_2,g_ as the carbon source.

Synthesis affinities of glycolic and pyruvic acids show a similar trend to the above-mentioned carboxylic acids, i.e., decrease at high temperatures and low pHs (Figure 4). However, the affinities are overall smaller than those of acetic and propanoic acids, respectively, and become negative at relatively lower temperatures (>140°C for glycolic acid and >220°C for pyruvic acid compared with >330°C for acetic acid and >310°C for propanoic acid, at pH = 7). Moreover, pH seems to have a more pronounced effect on the synthesis affinities of glycolic and pyruvic acids than acetic and propanoic acids.

### 3.3. Amino acids

**FIGURE 5:**
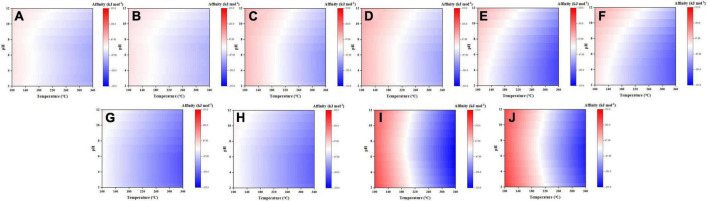
Synthesis affinity of glycine **(A,B)**, alanine **(C,D)**, aspartic acid **(E,F)**, serine **(G,H)**, and lysine **(I,J)** using CO,_g_ **(A,C,E,G,I)** and CO_2,g_ **(B,D,F,H,J)** as the carbon source.

Here, synthesis affinities of five selected amino acids (to reach 10^–6^ molal) were plotted as functions of temperature and pH (Figure 5), with the rest displayed in the Supplementary Figures 3–6. Among them, glycine, alanine, aspartic acid, and serine are major amino acids found in meteorites (Aponte et al., 2020; Glavin et al., 2020), and lysine, despite trace or no detection in meteorites, represents another type of amino acid with two amine groups. These five amino acids have overall negative synthesis affinities at elevated temperatures and the affinity increases at lower temperatures. Among them, the affinity of serine turns positive at temperature close to 100°C and pH > 7, whereas other four at temperature <180°C. For these five amino acids, the effect of pH on their affinities varies slightly: for glycine, alanine, and serine, their affinity increases at alkaline pHs but remains constant at acidic pHs; for aspartic acid, its affinity increases steadily from acidic pHs to alkaline pHs; for lysine, the affinity increases when pH increases or decreases with the minimal value at pH ∼7, possibly reflecting joint effects of –NH_2_ and –COOH groups.

**FIGURE 6:**
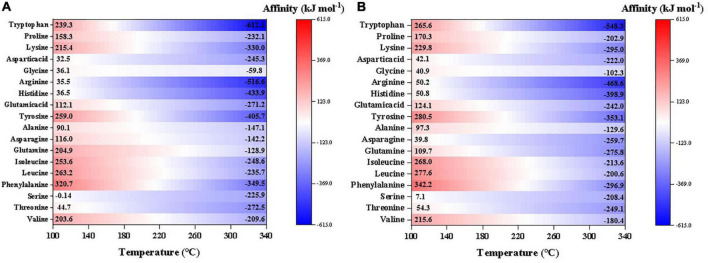
Synthesis affinity of proteinogenic amino acids excluding S-containing species using **(A)** CO,_g_ and **(B)** CO_2,g_ as the carbon source. In the calculations, pH was set neutral, which is also temperature-dependent.

For better comparison and presentation purposes, we have calculated and compared the synthesis affinities of 18 proteinogenic amino acids excluding S-containing species at neutral pH (temperature dependent, calculated by DEW model as well; Supplementary Figure 1). The result shows that the synthesis affinities decrease with increasing temperature and nearly all amino acids have negative synthesis affinities at T > 220°C (Figure 6). At T < 220°C, however, most of the amino acids have positive synthesis affinities with the order: phenylalanine > leucine∼isoleucine∼glutamine > valine∼tyrosine > tryptophan∼lysine > ∼proline∼glutamic acid∼alanine ∼asparagine > aspartic acid∼glycine∼threonine. In contrast, serine, histidine, and arginine have negative synthesis affinities across a large range of temperatures. Like other organics mentioned above, synthesis affinities values using CO as the carbon source are slightly lower than CO_2_.

### 3.4. Sugar, nucleobases, and nucleosides

**FIGURE 7:**
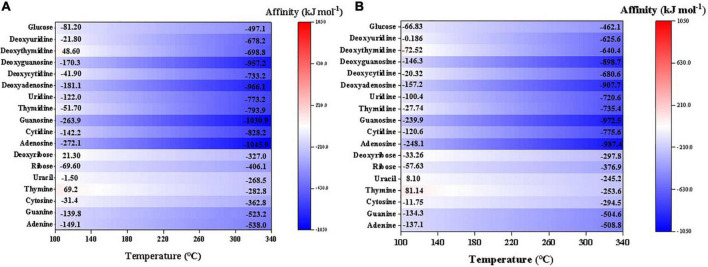
Synthesis affinity of sugar, nucleobases and nucleosides using **(A)** CO,_g_ and **(B)** CO_2,g_ as carbon source.

Nearly all the investigated sugar, nucleobases, and nucleosides have negative synthesis affinities (to reach 10^–6^ molal) at a wide range of temperature and the affinities increase with lower temperatures (Figure 7). Among them, deoxythymidine, deoxyribose, and thymine have positive affinities at relatively low temperatures, i.e., <140°C. Generally, nucleobases and sugars have similar synthesis affinity values, which are higher overall than the affinities of nucleosides. Like other organics, the affinities have slightly lower values using CO than CO_2_ as the C source.

## 4. Discussion and implications

### 4.1. Sensitivity of organic synthesis affinity to environment settings

Given that the abiotic synthesis reactions are essentially reduction of inorganic carbon (either CO_2_ or CO) by hydrogen, the hydrogen fugacity in the steam atmosphere would be expected to significantly affect the synthesis affinities. The above affinities were calculated by assuming the redox state constrained by a fixed partial pressure of H_2,*g*_ modeled by Zahnle et al. (2020) (Supplementary Figure 7). However, as also discussed in previous studies, the hydrogen pressure in the steam atmosphere might vary significantly depending on the composition of the impactor as well as the size of the impactor relatively to the ocean volume (i.e., reductant/oxidant flux ratio). More reducing impactor objects, like iron meteorites, would generate (at least locally) a more reducing atmosphere with higher hydrogen levels (Parkos et al., 2018; Zahnle et al., 2020; Pearce et al., 2022). Moreover, big impactors would consume more oxidants in the surface environments and induce larger-scale and longer-term reducing atmosphere (Segura et al., 2002; Parkos et al., 2018; Zahnle et al., 2020). In extreme cases, if pretty big impactors deplete the oxidants in the surface environments, the hydrogen level in the post-impact atmosphere might be predominantly buffered by the reducing mineral assemblages in the impactor, e.g., Fayalite (Fe_2_SiO_4_)-Quartz (SiO_2_)-Iron (Fe) (FQI) or Iron (Fe)-Wüstite (FeO) (IW) or else (Zahnle et al., 2020).

**FIGURE 8:**
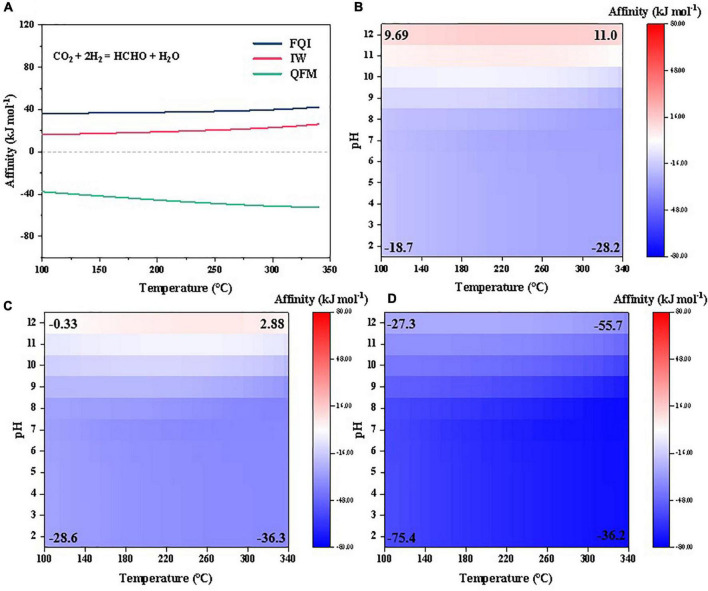
Synthesis affinity of **(A)** formaldehyde, **(B–D)** cyanide at different redox states [**(B)**, FQI; **(C)**, IW; **(D)**, FMQ] using CO_2_ as the carbon source.

**FIGURE 9:**
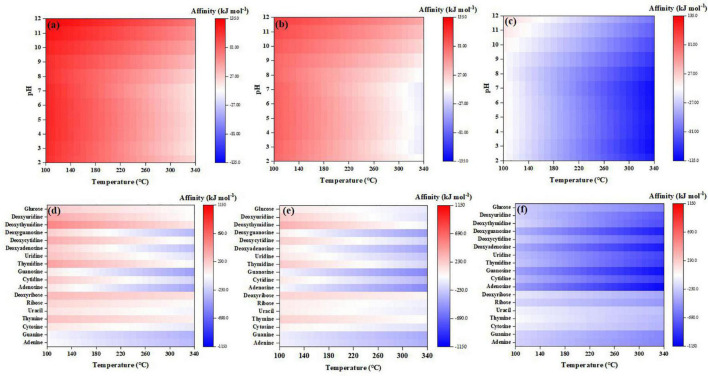
Synthesis affinity of **(a–c)** glycine, **(d–f)** sugars, nucleobases, and nucleosides at different redox states [**(a,d)**, FQI; **(b,e)** IW; **(c,f)**, FMQ] using CO_2_ as the carbon source.

Here, we calculated the partial pressure of H_2,*g*_ constrained by the equilibria of FQI and IW (Supplementary Figure 7) and then, examined the abiotic synthesis affinities of the selected organics using CO_2_ as the C source under various hydrogen fugacities (Figures 8, 9). We also compared the results with Fayalite (Fe_2_SiO_4_)-Magnetite (Fe_3_O_4_)-Quartz (SiO_2_) (FMQ) buffer, a more oxidizing mineral assemblage relevant with Earth’s differentiated mantle (Trail et al., 2011) and hydrothermal vent systems (Trail and McCollom, 2023). Our results suggest that the affinities are indeed highly sensitive to the hydrogen fugacity in the atmosphere, and higher hydrogen fugacity greatly favors synthesis of the studied compounds. For example, formaldehyde, which has negative synthesis affinities in a wide range of temperatures under the referenced redox condition (Figure 2C), becomes thermodynamically favored to form and accumulate (to >10^–6^ molal) at high hydrogen fugacities constrained by FQI and IW (Figure 8A). Similarly, abiotic synthesis of 10^–6^ molal cyanide becomes favorable at high hydrogen fugacities (FQI and IW) and high pH (>11) (Figures 8B, C). Glycine—which has the lowest synthesis affinity among amino acids studied under reference redox condition (Figure 6)–becomes strongly favored to form and accumulate to >10^–6^ molal under a wide range of temperatures and pHs at high hydrogen fugacities (FQI and IW; Figures 9A, B); all amino acids can therefore be expected to have strongly positive synthesis affinities at high hydrogen fugacities. Similarly, abiotic synthesis of most nucleosides, sugars and nucleobases (to 10^–6^ molal) becomes thermodynamically favorable at high hydrogen fugacities constrained by FQI and IW, except for adenine, guanine, adenosine and guanosine (Figures 9D, E).

However, at relatively low hydrogen fugacity constrained by FMQ, all the life building blocks considered in Figures 8, 9 have lower synthesis affinities compared with reference hydrogen fugacity, and are thus less favorable to form/accumulate. This redox state is close to the one in some hydrothermal systems on the Hadean Earth (Trail and McCollom, 2023), which was proposed to be one candidate environment for the emergence of life. Although we have not fully considered possibly different water chemistry in the hydrothermal systems, our results agree roughly with the early proposal that amino acids are not thermodynamically favorable to accumulate to levels higher than 10^–8^ molal in natural hydrothermal fluids (Amend and Shock, 1998). Thus, if the organics were synthesized locally around the crater(s) in the primitive ocean and diffused along the concentration gradient to the open ocean, they might tend to be degraded in the more oxidizing hydrothermal vents, which is the major sink for organic matter in the modern ocean (Lang et al., 2006). Previous studies once proposed that the mixing between hydrothermal fluid and seawater would favor the abiotic synthesis of some biomolecules (e.g., Shock and Schulte, 1998; Shock and Canovas, 2010; Amend et al., 2013). A re-evaluation of this hypothesis under primitive settings is complicated given the unknown fluid chemistries and beyond the scope of our study here. Above all, reducing capacity (i.e., hydrogen) of the post-impact atmosphere has a significant effect on the synthesis affinities of organics in the primitive Earth, and beneath a more reducing steam atmosphere (at FQI or IW buffer), nearly all of life building blocks are favorable to form.

The post-impact water-rock interaction would not only induce a reducing atmosphere, but also elevate the water pH (Kadoya et al., 2020) and generate secondary minerals (Schoonen and Smirnov, 2016) inside and around the craters. Our calculations suggest a positive effect of pH on the synthesis affinity from neutral to alkaline pHs. Abiotic synthesis of cyanide even becomes thermodynamically favorable at pH > 11 under more reducing atmospheres (Figures 8B–D). Such alkaline and reducing settings might be achieved in the surface waters around the craters. Our findings are also consistent with the previous proposals suggesting that alkaline waters were favorable for the accumulation of cyanide on primitive Earth (Toner and Catling, 2019). Moreover, previous studies also suggested that mineral adsorption could enhance the stability of life building blocks against potential threats like heating or UV radiation on the primitive Earth (Mignon et al., 2009; Hazen and Sverjensky, 2010; Grégoire et al., 2016). Such minerals are common product of the aqueous alteration of impactors (Rubin and Ma, 2017) and might enhance the stability of the abiotically synthesized building blocks of life against UV radiation in the shallow areas.

### 4.2. Stability of critical building blocks of life beneath the impact-induced steam atmosphere

Origin of life requires the accumulation of various inorganic and organic species as building blocks for chemical evolution. For example, HCN and HCHO were proposed to be the critical starting materials for the synthesis of nucleobases and sugar (Orgel, 1998; Yadav et al., 2020) and many origin-of-life experiments/hypotheses rely on high concentrations of these two compounds (Sanchez et al., 1967; Patel et al., 2015; Pérez-Fernández et al., 2022). Previous studies have proposed various pathways to form HCN and HCHO (Cleaves, 2008; Toner and Catling, 2019). Among them, the largest source was evaluated to be gaseous photochemistry in primitive atmosphere (Cleaves, 2008; Ferus et al., 2017; Pearce et al., 2022), especially after impact events (Parkos et al., 2018; Benner et al., 2020; Zahnle et al., 2020), when a more reducing atmosphere greatly improves synthesis efficiency (Tian et al., 2011; Pearce et al., 2022). Then, those photochemically generated building blocks of life would precipitate out of the steam atmosphere and accumulate in the ocean(s) for polymerization reactions (Benner et al., 2020).

However, according to our calculations, it is thermodynamically unfavorable to synthesize/accumulate appreciable levels (>10^–6^ molal) of HCN and HCHO, beneath a steam atmosphere after the impacts in the reference atmospheric composition. HCN is favored to form at higher hydrogen fugacities, which is also true for HCHO if the water is highly alkaline. Rainout fluxes of HCN and HCHO synthesized by photochemistry or lightning in the atmosphere would also compete with hydrolysis in surface waters; the steady-state level of HCN and HCHO would depend on the relative rates of rainout and hydrolysis. Previous experiments demonstrated rapid hydrolysis of HCN (Miyakawa et al., 2002) and HCHO (Kopetzki and Antonietti, 2011), with half-life times that are less than a day temperatures above 100°C. Previous studies have reported rainout flux of HCN in the post-impact steam atmosphere as 10^12^–10^14^ molecules/m^2^/s (Stribling and Miller, 1987; Parkos et al., 2018; Zahnle et al., 2020). Given the hydrolysis kinetics data of HCN from Miyakawa et al. (2002), the steady-state level of HCN in the surface water would be <10^–9^ molal at 100°C and pH = 6 (<10^–13^ molal at 200°C and pH = 6; higher temperatures would imply faster hydrolysis and thus lower steady-state concentrations of HCN). These levels of HCN are several orders of magnitude lower than the concentrations used in prebiotic synthesis experiments, e.g., concentrations of 10^–2^ molal and above used previously (Sanchez et al., 1967; Yadav et al., 2020; Pérez-Fernández et al., 2022). These results echo the previous suggestion that abiotic synthesis of biomolecules (amino and hydroxy acids or nucleosides) would be primarily constrained by the availability of starting bases including HCN and aldehydes in hot fluids (Schulte and Shock, 1995), even beneath a reducing steam-atmosphere after impacts. Meanwhile, strongly reducing atmospheres induced by large and more reducing impactors might favor the accumulation of critical bases for the synthesis reactions. Water bodies with high evaporation rates on land could also favor molecular condensation (Pérez-Fernández et al., 2022), but would be confronted with photodegradation processes (Cleaves, 2008; Todd et al., 2022).

### 4.3. Implications for life-searching efforts on other planetary bodies

In the solar system, Mars and some icy moons are believed to have (or have had) liquid water bodies and thus, represent the most promising places when looking for signs of extant or past alien life. An important target for life-searching efforts on these planetary bodies is organics, especially biomolecules. Mars, due to its proximity to the asteroid belts and its thin atmosphere, has been bombarded more heavily than Earth during its history (Hartmann and Neukum, 2001; Cox et al., 2022), and impactors might have delivered considerable fluxes of organics to its surface (Frantseva et al., 2018). Many craters are well preserved on Mars and have been selected in various landing missions as targets for *in situ* exploration. Our results suggest that some key building blocks of life, including HCN, HCHO, some amino acids, and nearly all nucleosides and sugars, could not be thermodynamically favorable to accumulate to a considerable level (>10^–6^ molal) for chemical evolution under relatively oxidizing conditions, but more reducing impactors together with alkaline conditions would favor abiotic synthesis and accumulation of most building blocks of life. These results may not be directly applicable to early Mars where crustal and atmospheric compositions were possibly different from early Earth, however, the general trend observed here should hold true. Direct observations of impactor material on the surface of planetary bodies through remote sensing methods are scarce since impactors cannot be well preserved in high-velocity impacts that create impact craters and basins. However, searching for reduced impactor material is possible *via in situ* imaging and measurements of the elemental or isotopic signatures. Here, we propose that during current and future Martian missions, the mission team should consider using the rover payload to (1) investigate impacted materials with likely more reducing impactor and (2) search for alkaline mineral assemblages that could be inducive of the accumulation and preservation of organics or biosignatures.

There are several icy moons in the solar system that show strong signs of the presence of subsurface ocean, such as Enceladus, Europa, Ganymede, and Titan (National Academies of Sciences, Engineering, and Medicine, 2022). Even more excitingly, the flyby analyses of the Enceladus plume revealed signals of various organics (Waite et al., 2009), including some complex O- and N-bearing species (Magee and Waite, 2017; Postberg et al., 2018). These observations place Enceladus among the most probably planetary bodies to find extraterrestrial life. There are also studies suggesting ongoing hydrothermal activity in the subsurface ocean of Enceladus (Hsu et al., 2015) and the hydrothermal heat flux might have been stronger in the past considering the progressive cooling of its core. However, the redox state of the hydrothermal systems on early Enceladus was suggested to be close to the FMQ buffer (Glein et al., 2008), and our current results suggest that formaldehyde, HCN, some amino acids, and nucleobases are not thermodynamically favorable to accumulate (>10^–6^ molal) under these conditions, even at the high pHs proposed for Enceladus’ ocean (Glein et al., 2018). Considering that Enceladus ocean water contains lower levels of other reactants involved in the synthesis reactions (i.e., CO_2_ and N_2_) (Waite et al., 2009; Glein et al., 2018), our results for the early Earth should be applicable to the Enceladus ocean as well. Given that Enceladus lacks an atmospheric source for these molecular compounds, they would tend to be completely degraded given the 100 s Ma to four Ga proposed history of Enceladus ocean (Ćuk et al., 2016; Neveu and Rhoden, 2019), as recently shown in a kinetic study about amino acids (Truong et al., 2019). Therefore, *in situ* origin of life in the subsurface ocean of icy moons might be threatened by the continuous hydrolysis of critical building blocks for the synthesis of biomolecules like sugar and nucleotide, in the hydrothermal systems.

## 5. Conclusion

In this study, we conducted thermodynamic calculations to evaluate the potential of abiotic synthesis of various building blocks of life beneath a simulative post-impact steam atmosphere. Our results show that the abiotic synthesis affinities generally decrease at elevated temperatures, and using CO as the C source would result in slightly higher synthesis affinities. Among the investigated compounds, many species, including cyanide, formaldehyde, several amino acids (e.g., aspartic acid, serine, and histidine), sugars, nucleosides, and nucleobases are not thermodynamically favored to form/accumulate to appreciable levels (>10^–6^ molal) in a wide range of temperatures. However, other organics would have positive affinities, especially in temperatures lower than 220°C and/or alkaline pHs. Our sensitivity tests also suggested that the reducing capacity of the atmosphere would have a significant effect on the abiotic synthesis: at higher hydrogen fugacity in equilibrium with FQI or IW buffers, abiotic synthesis of nearly all building blocks of life (except HCN, adenine, guanine, adenosine and guanosine) becomes thermodynamically favorable, but at lower hydrogen fugacity in equilibrium with FMQ, the abiotic synthesis becomes less favorable than under the reference redox conditions. Thus, although impact events could induce a hydrogen-rich atmosphere, accumulation of some critical building blocks of life, e.g., HCN, formaldehyde, and their derivates, and might still be hard to achieve, unless in an atmosphere induced by a very large and reducing impactor. Finally, we suggest that craters generated by more reducing impactors could potentially be ideal sites in the search for biomolecules and biosignatures on other planets like Mars.

## Data availability statement

The original contributions presented in this study are included in the article/Supplementary material, further inquiries can be directed to the corresponding author.

## Author contributions

JH designed the research. ZZ, HJ, PJ, LP, and JH conducted the research. GZ involved in the writing and revision of the manuscript. All authors discussed the results and wrote the manuscript and approved the submitted version.

## Footnotes

1
http://www.dewcommunity.org/

## References

[B1] Amend J. P. LaRowe D. E. McCollom T. M. Shock E. L. (2013). The energetics of organic synthesis inside and outside the cell. *Philos. Trans. R. Soc. B Biol. Sci.* 368:20120255. 10.1098/rstb.2012.0255 23754809 PMC3685458

[B2] Amend J. P. Shock E. L. (1998). Energetics of amino acid synthesis in hydrothermal ecosystems. *Science* 281 1659–1662. 10.1126/science.281.5383.1659 9733509

[B3] Aponte J. C. Elsila J. E. Hein J. E. Dworkin J. P. Glavin D. P. McLain H. L. (2020). Analysis of amino acids, hydroxy acids, and amines in CR chondrites. *Meteorit. Planet. Sci.* 55 2422–2439. 10.1111/maps.13586 33536738 PMC7839561

[B4] Bar-Nun A. Bar-Nun N. Bauer S. H. Sagan C. (1970). Shock synthesis of amino acids in simulated primitive environments. *Science* 168 470–473. 10.1126/science.168.3930.470 5436082

[B5] Benner S. A. Bell E. A. Biondi E. Brasser R. Carell T. Kim H.-J. (2020). When did life likely emerge on earth in an RNA-first process? *ChemSystemsChem* 2:e1900035. 10.1002/syst.201900035

[B6] Cardace D. Meyer-Dombard D. R. Woycheese K. M. Arcilla C. A. (2015). Feasible metabolisms in high pH springs of the Philippines. *Front. Microbiol.* 6:10. 10.3389/fmicb.2015.00010 25713561 PMC4322734

[B7] Chyba C. Sagan C. (1992). Endogenous production, exogenous delivery and impact-shock synthesis of organic molecules: an inventory for the origins of life. *Nature* 355 125–132. 10.1038/355125a0 11538392

[B8] Cleaves H. J. (2008). The prebiotic geochemistry of formaldehyde. *Precambrian Res.* 164 111–118. 10.1016/j.precamres.2008.04.002

[B9] Cox M. A. Cavosie A. J. Orr K. J. Daly L. Martin L. Lagain A. (2022). Impact and habitability scenarios for early mars revisited based on a 4.45-Ga shocked zircon in regolith breccia. *Sci. Adv.* 8:eabl7497. 10.1126/sciadv.abl7497 35108046 PMC8809541

[B10] Cronin J. R. Chang S. (1993). “Organic matter in meteorites: molecular and isotopic analyses of the murchison meteorite BT - the chemistry of life’s origins,” in *The Chemisty of Life’s Origins*, eds Greenberg J. M. Mendoza-Gómez C. X. Pirronello V. (Dordrecht: Springer) 10.1007/978-94-011-1936-8_9

[B11] Ćuk M. Dones L. Nesvorný D. (2016). Dynamical evidence for a late formation of Saturn’S moons. *Astrophys. J.* 820:97. 10.3847/0004-637X/820/2/97

[B12] Ferus M. Kubelík P. Knížek A. Pastorek A. Sutherland J. Civiš S. (2017). High energy radical chemistry formation of HCN-rich atmospheres on early Earth. *Sci. Rep.* 7:6275. 10.1038/s41598-017-06489-1 28740207 PMC5524942

[B13] Feulner G. (2012). The faint young sun problem. *Rev. Geophys.* 50, 1–32. 10.1029/2011RG000375

[B14] Franiatte M. Richard L. Elie M. Nguyen-Trung C. Perfetti E. LaRowe D. E. (2008). Hydrothermal stability of adenine under controlled fugacities of N2. CO2 and H2. *Orig. Life Evol. Biosph.* 38 139–148. 10.1007/s11084-008-9126-5 18297413

[B15] Frantseva K. Mueller M. ten Kate I. L. van der Tak F. F. S. Greenstreet S. (2018). Delivery of organics to Mars through asteroid and comet impacts. *Icarus* 309 125–133. 10.1016/j.icarus.2018.03.006

[B16] Furukawa Y. Chikaraishi Y. Ohkouchi N. Ogawa N. O. Glavin D. P. Dworkin J. P. (2019). Extraterrestrial ribose and other sugars in primitive meteorites. *Proc. Natl. Acad. Sci.* 116 24440–24445. 10.1073/pnas.1907169116 31740594 PMC6900709

[B17] Furukawa Y. Sekine T. Oba M. Kakegawa T. Nakazawa H. (2009). Biomolecule formation by oceanic impacts on early earth. *Nat. Geosci.* 2 62–66. 10.1038/ngeo383

[B18] Genda H. Brasser R. Mojzsis S. J. (2017a). The terrestrial late veneer from core disruption of a lunar-sized impactor. *Earth Planetary Sci. Lett.* 480 25–32. 10.1016/j.epsl.2017.09.041

[B19] Genda H. Iizuka T. Sasaki T. Ueno Y. Ikoma M. (2017b). Ejection of iron-bearing giant-impact fragments and the dynamical and geochemical influence of the fragment re-accretion. *Earth Planet. Sci. Lett.* 470 87–95. 10.1016/j.epsl.2017.04.035

[B20] Glavin D. P. McLain H. L. Dworkin J. P. Parker E. T. Elsila J. E. Aponte J. C. (2020). Abundant extraterrestrial amino acids in the primitive CM carbonaceous chondrite Asuka 12236. *Meteorit. Planet. Sci.* 55 1979–2006. 10.1111/maps.13560

[B21] Glein C. R. Postberg F. Vance S. D. (2018). “The geochemistry of enceladus: composition and controls,” in *Enceladus and the Icy Moons of Saturn*, eds Schenk P. M. Clark R. N. Howett C. J. A. (Tucson, AZ: University of Arizona Press). 10.2458/azu_uapress_9780816537075-ch003

[B22] Glein C. R. Zolotov M. Y. Shock E. L. (2008). The oxidation state of hydrothermal systems on early Enceladus. *Icarus* 197 157–163. 10.1016/j.icarus.2008.03.021

[B23] Goesmann F. Rosenbauer H. Bredehöft J. H. Cabane M. Ehrenfreund P. Gautier T. (2015). Organic compounds on comet 67P/Churyumov-Gerasimenko revealed by COSAC mass spectrometry. *Science* 349:aab0689. 10.1126/science.aab0689 26228156

[B24] Goldman N. Reed E. J. Fried L. E. William Kuo I. F. Maiti A. (2010). Synthesis of glycine-containing complexes in impacts of comets on early Earth. *Nat. Chem.* 2 949–954. 10.1038/nchem.827 20966951

[B25] Grégoire B. Erastova V. Geatches D. L. Clark S. J. Greenwell H. C. Fraser D. G. (2016). Insights into the behaviour of biomolecules on the early Earth: the concentration of aspartate by layered double hydroxide minerals. *Geochimica et Cosmochimica Acta* 176 239–258. 10.1016/j.gca.2015.12.026

[B26] Gulmann L. K. Beaulieu S. E. Shank T. M. Ding K. Seyfried W. E. Sievert S. M. (2015). Bacterial diversity and successional patterns during biofilm formation on freshly exposed basalt surfaces at diffuse-flow deep-sea vents. *Front. Microbiol.* 6:901. 10.3389/fmicb.2015.00901 26441852 PMC4564720

[B27] Haldane J. B. S. (1929). The origin of life. *Rationalist Annu.* 148 3–10.

[B28] Hao J. Sverjensky D. A. Hazen R. M. (2019). Redox states of Archean surficial environments: the importance of H2,g instead of O2,g for weathering reactions. *Chem. Geol.* 521 49–58. 10.1016/j.chemgeo.2019.05.022

[B29] Hartmann W. K. Neukum G. (2001). Cratering chronology and the evolution of Mars. *Space Sci. Rev.* 96 165–194. 10.1023/A:1011945222010

[B30] Hashimoto G. L. Abe Y. Sugita S. (2007). The chemical composition of the early terrestrial atmosphere: formation of a reducing atmosphere from CI-like material. *J. Geophys. Res. Planets* 112:E05010. 10.1029/2006JE002844

[B31] Hayes J. M. (1967). Organic constituents of meteorites—a review. *Geochimica et Cosmochimica Acta* 31 1395–1440. 10.1016/0016-7037(67)90019-1 4892655

[B32] Hazen R. M. Sverjensky D. A. (2010). Mineral surfaces, geochemical complexities, and the origins of life. *Cold Spring Harb. Perspect. Biol.* 2:a002162. 10.1101/cshperspect.a002162 20452963 PMC2857174

[B33] Hsu H.-W. Postberg F. Sekine Y. Shibuya T. Kempf S. Horányi M. (2015). Ongoing hydrothermal activities within Enceladus. *Nature* 519 207–210. 10.1038/nature14262 25762281

[B34] Kadoya S. Krissansen-Totton J. Catling D. C. (2020). Probable cold and alkaline surface environment of the hadean earth caused by impact ejecta weathering. *Geochem. Geophys. Geosyst.* 21:e2019GC008734. 10.1029/2019GC008734

[B35] Kasting J. F. (1993). Earth’s early atmosphere. *Science* 259 920–926. 10.1126/science.11536547 11536547

[B36] Kasting J. F. (2014). “Atmospheric composition of hadean–early Archean Earth: the importance of CO,” in *Earth’s Early Atmosphere and Surface Environment*, ed. Shaw G. H. (Boulder, CO: Geological Society of America). 10.1130/2014.2504(04)

[B37] Kobayashi K. Kaneko T. Saito T. Oshima T. (1998). Amino acid formation in gas mixtures by high energy particle irradiation. *Origins Life Evol. Biosph.* 28 155–165. 10.1023/A:1006561217063 11536862

[B38] Kopetzki D. Antonietti M. (2011). Hydrothermal formose reaction. *New J. Chem.* 35 1787–1794. 10.1039/c1nj20191c

[B39] Kuramoto K. Umemoto T. Ishiwatari M. (2013). Effective hydrodynamic hydrogen escape from an early Earth atmosphere inferred from high-accuracy numerical simulation. *Earth Planet. Sci. Lett.* 375 312–318. 10.1016/j.epsl.2013.05.050

[B40] Kvenvolden K. Lawless J. Pering K. Peterson E. Flores J. Ponnamperuma C. (1970). Evidence for extraterrestrial amino-acids and hydrocarbons in the Murchison Meteorite. *Nature* 228 923–926. 10.1038/228923a0 5482102

[B41] Lang S. Q. Butterfield D. A. Lilley M. D. Paul Johnson H. Hedges J. I. (2006). Dissolved organic carbon in ridge-axis and ridge-flank hydrothermal systems. *Geochimica et Cosmochimica Acta* 70 3830–3842. 10.1016/j.gca.2006.04.031

[B42] Lazcano A. Miller S. L. (1994). How long did it take for life to begin and evolve to cyanobacteria? *J. Mol. Evol.* 39 546–554. 10.1007/BF00160399 11536653

[B43] Li Y. Li Y. Liu Y. Wu Y. Wu J. Wang B. (2022). Photoreduction of inorganic carbon(+IV) by elemental sulfur: implications for prebiotic synthesis in terrestrial hot springs. *Sci. Adv.* 6:eabc3687. 10.1126/sciadv.abc3687 33208363 PMC7673799

[B44] Lohrmann R. Orgel L. E. (1971). Urea-inorganic phosphate mixtures as prebiotic phosphorylating agents. *Science* 171 490–494. 10.1126/science.171.3970.490 5099649

[B45] Magee B. A. Waite J. H. (2017). *Neutral Gas Composition of Enceladus’ Plume-Model Parameter Insights from Cassini–INMS*. Lunar and Planetary Science XLVIII. 2974.

[B46] Martins Z. Price M. C. Goldman N. Sephton M. A. Burchell M. J. (2013). Shock synthesis of amino acids from impacting cometary and icy planet surface analogues. *Nat. Geosci.* 6 1045–1049. 10.1038/ngeo1930

[B47] McCollom T. M. Seewald J. S. (2007). Abiotic synthesis of organic compounds in deep-sea hydrothermal environments. *Chem. Rev.* 107 382–401. 10.1021/cr0503660 17253758

[B48] McDermott J. M. Seewald J. S. German C. R. Sylva S. P. (2015). Pathways for abiotic organic synthesis at submarine hydrothermal fields. *Proc. Natl. Acad. Sci.* 112 7668–7672. 10.1073/pnas.1506295112 26056279 PMC4485091

[B49] Ménez B. Pisapia C. Andreani M. Jamme F. Vanbellingen Q. P. Brunelle A. (2018). Abiotic synthesis of amino acids in the recesses of the oceanic lithosphere. *Nature* 564 59–63. 10.1038/s41586-018-0684-z 30405236

[B50] Mignon P. Ugliengo P. Sodupe M. (2009). Theoretical study of the adsorption of RNA/DNA bases on the external surfaces of Na+-montmorillonite. *J. Phys. Chem. C* 113 13741–13749. 10.1021/jp901699q 22124483

[B51] Miller S. L. (1953). A production of amino acids under possible primitive Earth conditions. *Science* 117 528–529. 10.1126/science.117.3046.528 13056598

[B52] Miyakawa S. James Cleaves H. Miller S. L. (2002). The cold origin of life: a. implications based on the hydrolytic stabilities of hydrogen cyanide and formamide. *Orig. Life Evol. Biosph.* 32 195–208. 10.1023/A:1016514305984 12227424

[B53] National Academies of Sciences, Engineering, and Medicine (2022). *Origins, Worlds, and Life: a Decadal Strategy for Planetary Science and Astrobiology 2023-2032.* Washington, DC: National Academies Press.

[B54] Neveu M. Rhoden A. R. (2019). Evolution of Saturn’s mid-sized moons. *Nat. Astron.* 3 543–552. 10.1038/s41550-019-0726-y 31360776 PMC6662725

[B55] Oba Y. Takano Y. Furukawa Y. Koga T. Glavin D. P. Dworkin J. P. (2022). Identifying the wide diversity of extraterrestrial purine and pyrimidine nucleobases in carbonaceous meteorites. *Nat. Commun.* 13:2008. 10.1038/s41467-022-29612-x 35473908 PMC9042847

[B56] Oparin A. I. Morgulis S. (1938). *Âîçíèêíîâåíèå Æèçíè Íà Çåìëå. The Origin of Life. Translation with Annotations by Sergius Morgulis.* New York, NY: Macmillan Company.

[B57] Orgel L. E. (1998). The origin of life—a review of facts and speculations. *Trends Biochem. Sci.* 23 491–495. 10.1016/S0968-0004(98)01300-0 9868373

[B58] Parkos D. Pikus A. Alexeenko A. Melosh H. J. (2018). HCN production via impact ejecta reentry during the late heavy bombardment. *J. Geophys. Res. Planets* 123 892–909. 10.1002/2017JE005393

[B59] Patel B. H. Percivalle C. Ritson D. J. Duffy C. D. Sutherland J. D. (2015). Common origins of RNA, protein and lipid precursors in a cyanosulfidic protometabolism. *Nat. Chem.* 7 301–307. 10.1038/nchem.2202 25803468 PMC4568310

[B60] Pearce B. K. D. Molaverdikhani K. Pudritz R. E. Henning T. Cerrillo K. E. (2022). Toward RNA life on early Earth: from atmospheric HCN to biomolecule production in warm little ponds. *Astrophys. J.* 932:9. 10.3847/1538-4357/ac47a1

[B61] Pérez-Fernández C. Vega J. Rayo-Pizarroso P. Mateo-Marti E. Ruiz-Bermejo M. (2022). Prebiotic synthesis of noncanonical nucleobases under plausible alkaline hydrothermal conditions. *Sci. Rep.* 12:15140. 10.1038/s41598-022-19474-0 36071125 PMC9452575

[B62] Pizzarello S. Cooper G. W. Flynn G. J. (2006). The nature and distribution of the organic material in carbonaceous chondrites and interplanetary dust particles. *Meteorites Early Solar Syst. II* 1 625–651. 10.2307/j.ctv1v7zdmm.36 11536464

[B63] Plyasunov A. V. Shock E. L. (2001). Correlation strategy for determining the parameters of the revised Helgeson-Kirkham-flowers model for aqueous nonelectrolytes. *Geochimica et Cosmochimica Acta* 65 3879–3900. 10.1016/S0016-7037(01)00678-0

[B64] Postberg F. Khawaja N. Abel B. Choblet G. Glein C. R. Gudipati M. S. (2018). Macromolecular organic compounds from the depths of Enceladus. *Nature* 558 564–568. 10.1038/s41586-018-0246-4 29950623 PMC6027964

[B65] Powner M. W. Gerland B. Sutherland J. D. (2009). Synthesis of activated pyrimidine ribonucleotides in prebiotically plausible conditions. *Nature* 459 239–242. 10.1038/nature08013 19444213

[B66] Rimmer P. B. Rugheimer S. (2019). Hydrogen cyanide in nitrogen-rich atmospheres of rocky exoplanets. *Icarus* 329 124–131. 10.1016/j.icarus.2019.02.020

[B67] Rubin A. E. Ma C. (2017). Meteoritic minerals and their origins. *Geochemistry* 77 325–385. 10.1016/j.chemer.2017.01.005

[B68] Russell M. J. Nitschke W. (2017). Methane: fuel or exhaust at the emergence of life? *Astrobiology* 17 1053–1066. 10.1089/ast.2016.1599 28949766 PMC5655419

[B69] Ryder G. (2002). Mass flux in the ancient Earth-Moon system and benign implications for the origin of life on Earth. *J. Geophys. Res. E Planets* 107 6–1. 10.1029/2001JE001583

[B70] Sanchez R. A. Ferbis J. P. Orgel L. E. (1967). Studies in prebiodc synthesis: II. synthesis of purine precursors and amino acids from aqueous hydrogen cyanide. *J. Mol. Biol.* 30 223–253. 4297187

[B71] Schaefer L. Fegley B. (2017). Redox states of initial atmospheres outgassed on rocky planets and planetesimals. *Astrophys. J.* 843:120. 10.3847/1538-4357/aa784f

[B72] Schlesinger G. Miller S. L. (1983). Prebiotic synthesis in atmospheres containing CH4. CO, and CO2. *J. Mol. Evol.* 19 383–390. 10.1007/BF02101643 6315963

[B73] Schoonen M. Smirnov A. (2016). Staging life in an early warm ‘Seltzer’. *Ocean. Elements* 12 395–400. 10.2113/gselements.12.6.395

[B74] Schulte M. Shock E. (1995). Thermodynamics of strecker synthesis in hydrothermal systems. *Orig. Life Evol. Biosph.* 25 161–173. 10.1007/BF01581580 11536668

[B75] Schulte M. D. Shock E. L. (1993). Aldehydes in hydrothermal solution: standard partial molal thermodynamic properties and relative stabilities at high temperatures and pressures. *Geochimica et Cosmochimica Acta* 57 3835–3846. 10.1016/0016-7037(93)90337-V 11539453

[B76] Seewald J. S. Reeves E. P. Bach W. Saccocia P. J. Craddock P. R. Shanks W. C. III (2015). Submarine venting of magmatic volatiles in the Eastern Manus Basin, Papua New Guinea. *Geochim. Cosmochim. Acta* 163, 178–199. 10.1111/1462-2920.13173 26663423 PMC5021209

[B77] Segura T. L. Toon O. B. Colaprete A. Zahnle K. (2002). Environmental effects of large impacts on Mars. *Science* 298 1977–1980. 10.1126/science.1073586 12471254

[B78] Shock E. Canovas P. (2010). The potential for abiotic organic synthesis and biosynthesis at seafloor hydrothermal systems. *Geofluids* 10 161–192. 10.1002/9781444394900.ch12

[B79] Shock E. L. (1988). Organic acid metastability in sedimentary basins. *Geology* 16 886–890. 10.1130/0091-7613(1988)016<0886:OAMISB>2.3.CO;2

[B80] Shock E. L. (1990). Do amino acids equilibrate in hydrothermal fluids? *Geochimica et Cosmochimica Acta* 54 1185–1189. 10.1016/0016-7037(90)90450-Y

[B81] Shock E. L. Helgeson H. C. (1990). Calculation of the thermodynamic and transport properties of aqueous species at high pressures and temperatures: standard partial molal properties of organic species. *Geochimica et Cosmochimica Acta* 54 915–945. 10.1016/0016-7037(90)90429-O

[B82] Shock E. L. Oelkers E. H. Johnson J. W. Sverjensky D. A. Helgeson H. C. (1992). Calculation of the thermodynamic properties of aqueous species at high pressures and temperatures. Effective electrostatic radii, dissociation constants and standard partial molal properties to 1000 °C and 5 kbar. *J. Chem. Soc. Faraday Trans.* 88 803–826. 10.1039/FT9928800803

[B83] Shock E. L. Sassani D. C. Willis M. Sverjensky D. A. (1997). Inorganic species in geologic fluids: correlations among standard molal thermodynamic properties of aqueous ions and hydroxide complexes. *Geochimica et Cosmochimica Acta* 61 907–950. 10.1016/S0016-7037(96)00339-0 11541225

[B84] Shock E. L. Schulte M. D. (1998). Organic synthesis during fluid mixing in hydrothermal systems. *J. Geophys. Res. Planets* 103 28513–28527. 10.1029/98JE02142

[B85] Stribling R. Miller S. L. (1987). Energy yields for hydrogen cyanide and formaldehyde syntheses: the hcn and amino acid concentrations in the primitive ocean. *Orig. Life Evol. Biosph.* 17 261–273. 10.1007/BF02386466 2819806

[B86] Sugahara H. Mimura K. (2015). Peptide synthesis triggered by comet impacts: a possible method for peptide delivery to the early Earth and icy satellites. *Icarus* 257 103–112. 10.1016/j.icarus.2015.04.029

[B87] Sverjensky D. A. Harrison B. Azzolini D. (2014). Water in the deep Earth: the dielectric constant and the solubilities of quartz and corundum to 60kb and 1200°C. *Geochimica et Cosmochimica Acta* 129 125–145. 10.1016/j.gca.2013.12.019

[B88] Takeuchi Y. Furukawa Y. Kobayashi T. Sekine T. Terada N. Kakegawa T. (2020). Impact-induced amino acid formation on Hadean Earth and Noachian Mars. *Sci. Rep.* 10:9220. 10.1038/s41598-020-66112-8 32513990 PMC7280214

[B89] Tian F. Kasting J. F. Zahnle K. (2011). Revisiting HCN formation in Earth’s early atmosphere. *Earth Planet. Sci. Lett.* 308 417–423. 10.1016/j.epsl.2011.06.011

[B90] Todd Z. R. Lozano G. G. Kufner C. L. Sasselov D. D. Catling D. C. (2022). Ferrocyanide survival under near ultraviolet (300–400 nm) irradiation on early Earth. *Geochimica et Cosmochimica Acta* 335 1–10. 10.1016/j.gca.2022.08.012

[B91] Toner J. D. Catling D. C. (2019). Alkaline lake settings for concentrated prebiotic cyanide and the origin of life. *Geochimica et Cosmochimica Acta* 260 124–132. 10.1016/j.gca.2019.06.031

[B92] Trail D. McCollom T. M. (2023). Relatively oxidized fluids fed Earth’s earliest hydrothermal systems. *Science* 379 582–586. 10.46427/gold2022.12094 36758072

[B93] Trail D. Watson E. B. Tailby N. D. (2011). The oxidation state of Hadean magmas and implications for early Earth’s atmosphere. *Nature* 480 79–82. 10.1038/nature10655 22129728

[B94] Truong N. Monroe A. A. Glein C. R. Anbar A. D. Lunine J. I. (2019). Decomposition of amino acids in water with application to in-situ measurements of Enceladus, Europa and other hydrothermally active icy ocean worlds. *Icarus* 329 140–147. 10.1016/j.icarus.2019.04.009

[B95] Waite J. H. Lewis W. S. Magee B. A. Lunine J. I. McKinnon W. B. Glein C. R. (2009). Liquid water on Enceladus from observations of ammonia and 40Ar in the plume. *Nature* 460 487–490. 10.1038/nature08153

[B96] Yadav M. Kumar R. Krishnamurthy R. (2020). Chemistry of abiotic nucleotide synthesis. *Chem. Rev.* 120 4766–4805. 10.1021/acs.chemrev.9b00546 31916751

[B97] Zahnle K. J. Lupu R. Catling D. C. Wogan N. (2020). Creation and evolution of impact-generated reduced atmospheres of early Earth. *Planet. Sci. J.* 1:11. 10.3847/PSJ/ab7e2c

[B98] Zang X. Ueno Y. Kitadai N. (2022). Photochemical synthesis of ammonia and amino acids from nitrous oxide. *Astrobiology* 22 387–398. 10.1089/ast.2021.0064 35196128

