# Supplementary material for: Evaluating the abiotic synthesis potential and the stability of building blocks of life beneath an impact-induced steam atmosphere
Source: Front Microbiol. 2023 Apr 3;14:1032073. doi: 10.3389/fmicb.2023.1032073 (PMC10116804; doi:10.3389/fmicb.2023.1032073)
Supplement: Supplementary file 1 [file Data_Sheet_1.docx]

Supplementary Material

# Supplementary Figures and Tables

## Figure S1


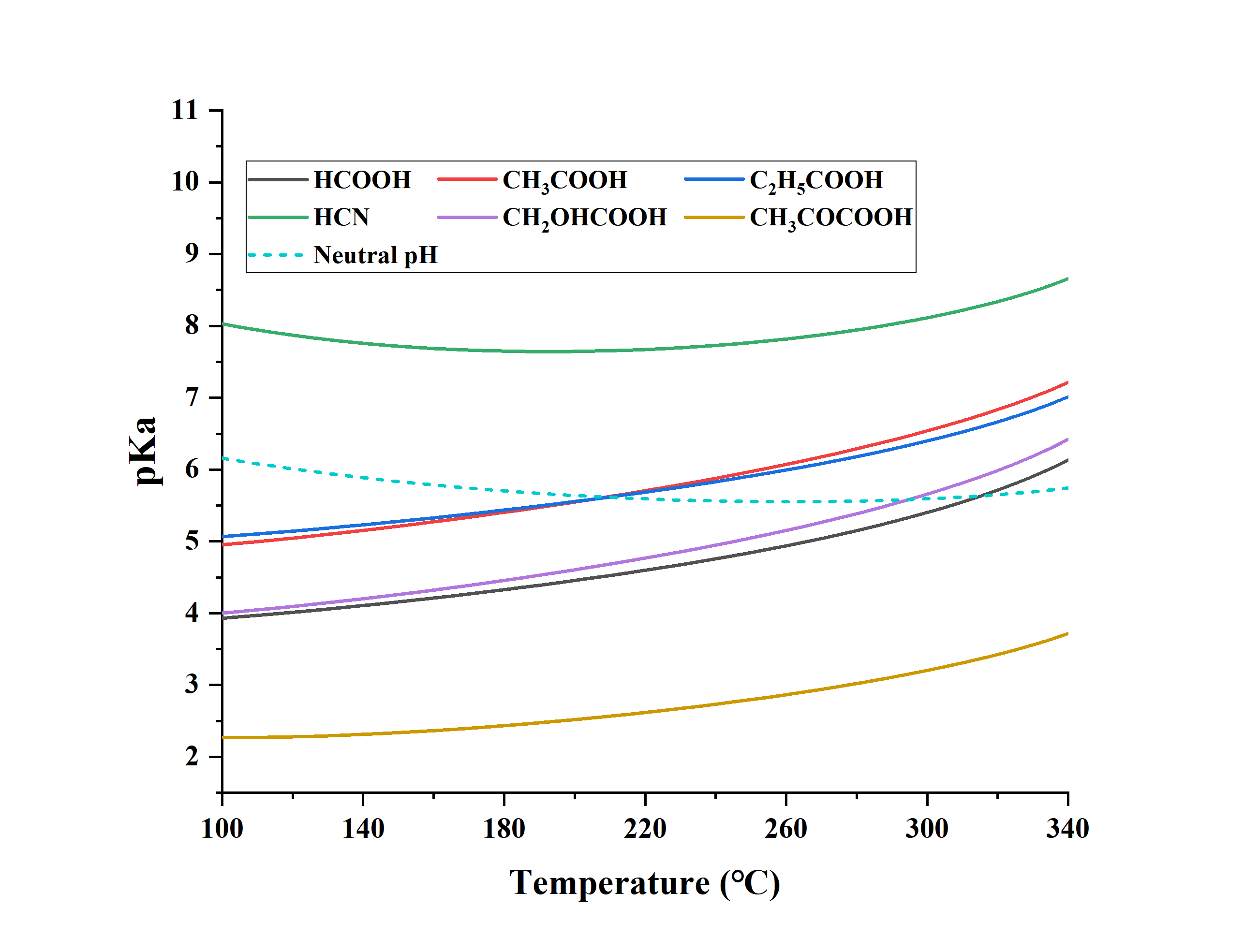


**Figure S1.** Evolution of pKa values for HCN and carboxylic acids as a function of temperature at water vapor saturation pressure; dashed line shows the neutral pH.

## Figures S2

**Figure S2.** Comparison of the equilibrium reaction constant and reaction quotient for: ${CO}_{2,g}+H_{2,g}\leftrightarrow{CO}_{g}+H_{2}O$. The reaction quotient was calculated using atmospheric composition in **Table 1**.

## Figures S3


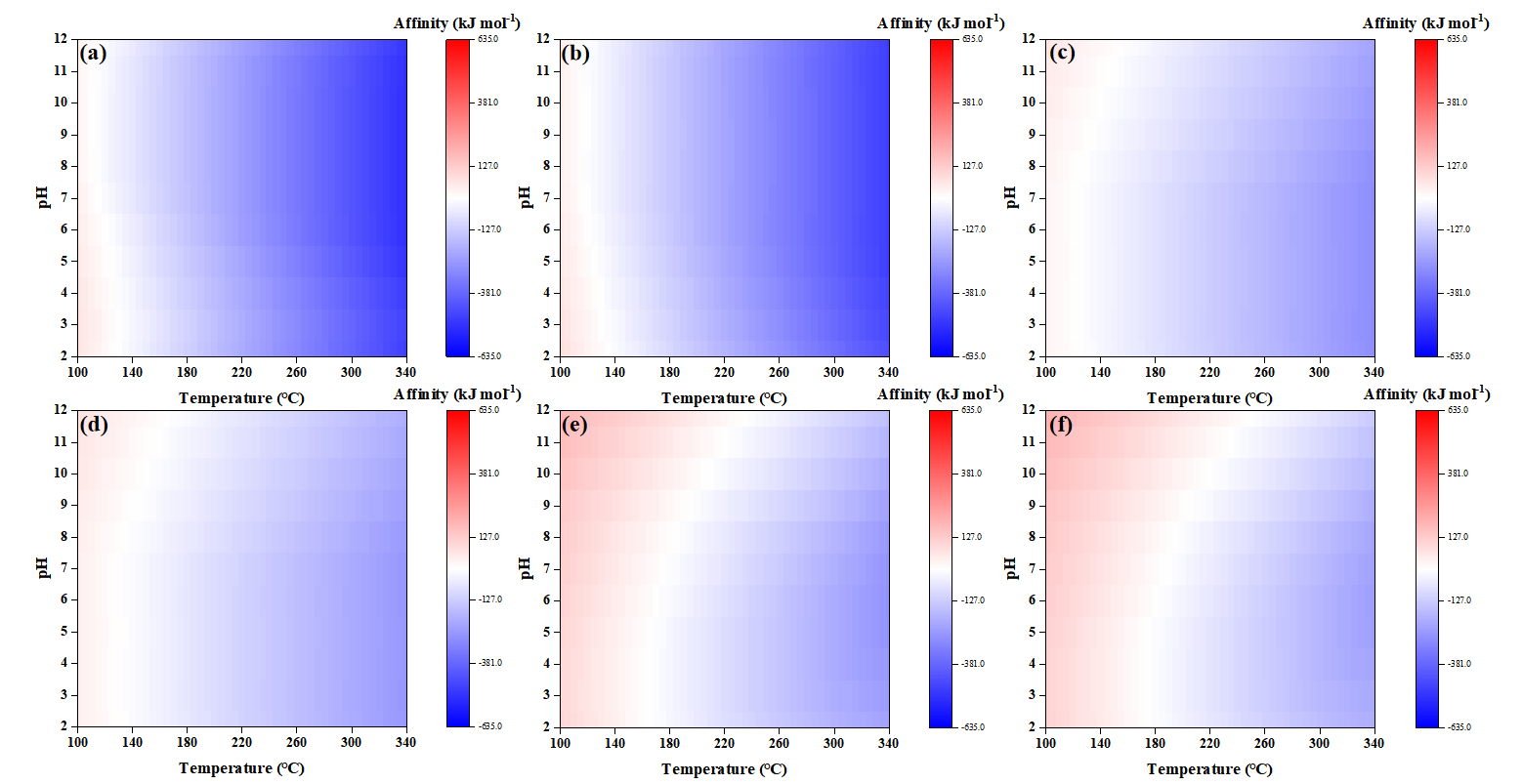


**Figure S3.** Synthesis affinity of arginine (a, b), asparagine (c, d), and glutamic acid (e, f) using (a, c, e) CO,_g_ and (b, d, f) CO_2_,_g_ as the carbon source.

## Figures S4


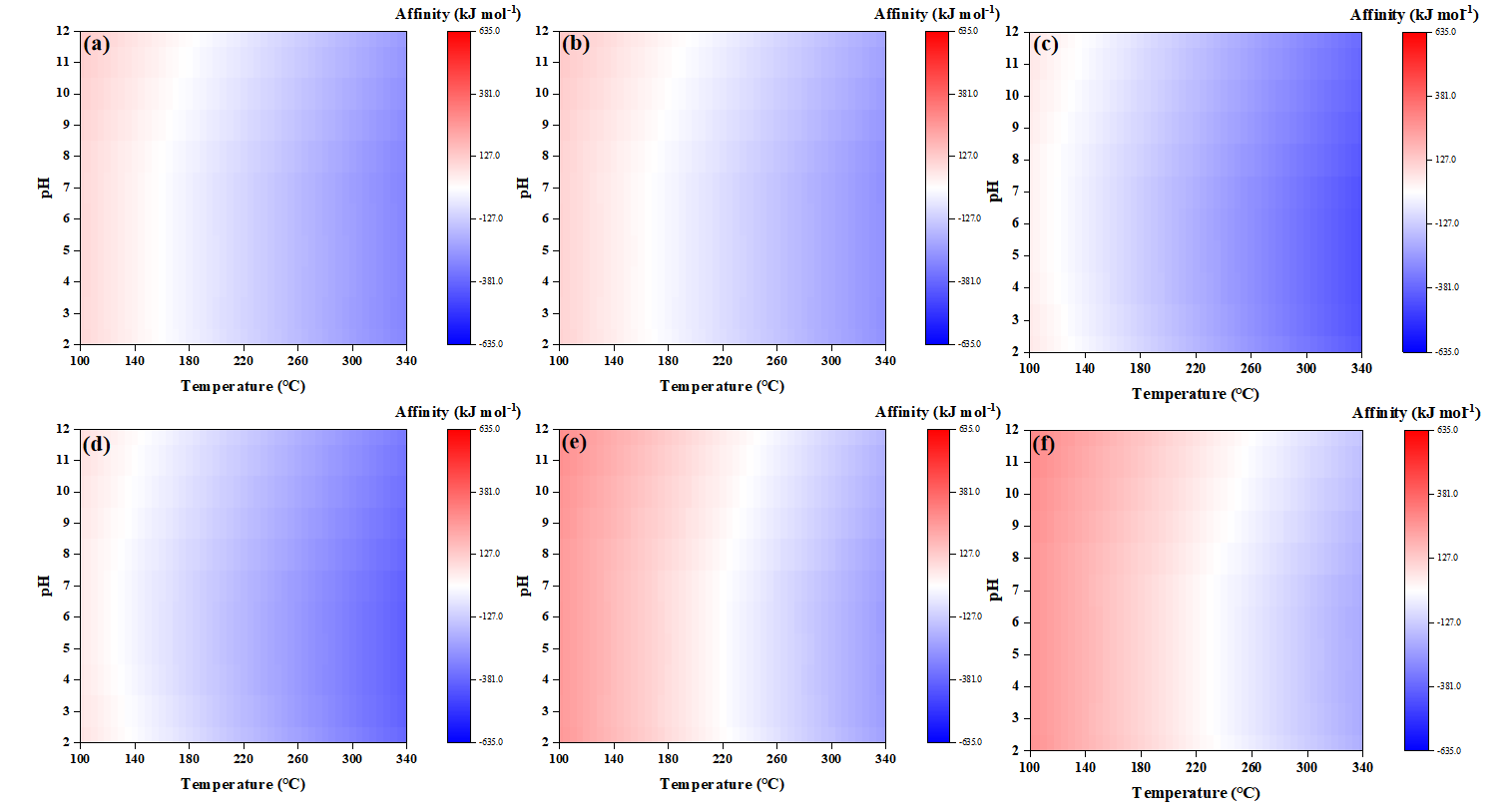


**Figure S4.** Synthesis affinity of glutamine (a, b), histidine (c, d), and isoleucine (e, f) using (a, c, e) CO,_g_ and (b, d, f) CO_2_,_g_ as the carbon source.

## Figures S5


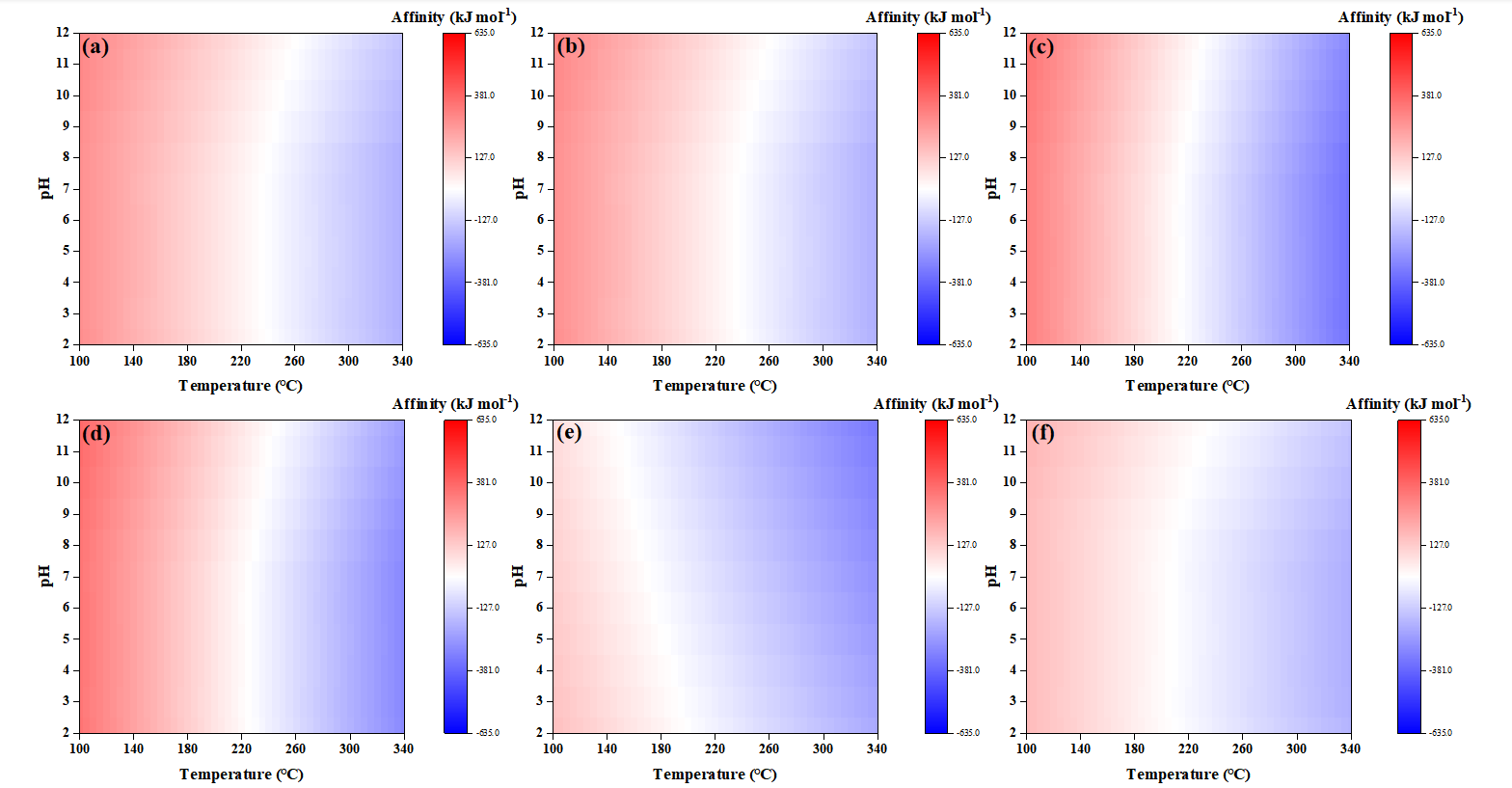


**Figure S5.** Synthesis affinity of leucine (a, b), phenylalanine (c, d), and proline (e, f) using (a, c, e) CO,_g_ and (b, d, f) CO_2_,_g_ as the carbon source.

## Figures S6


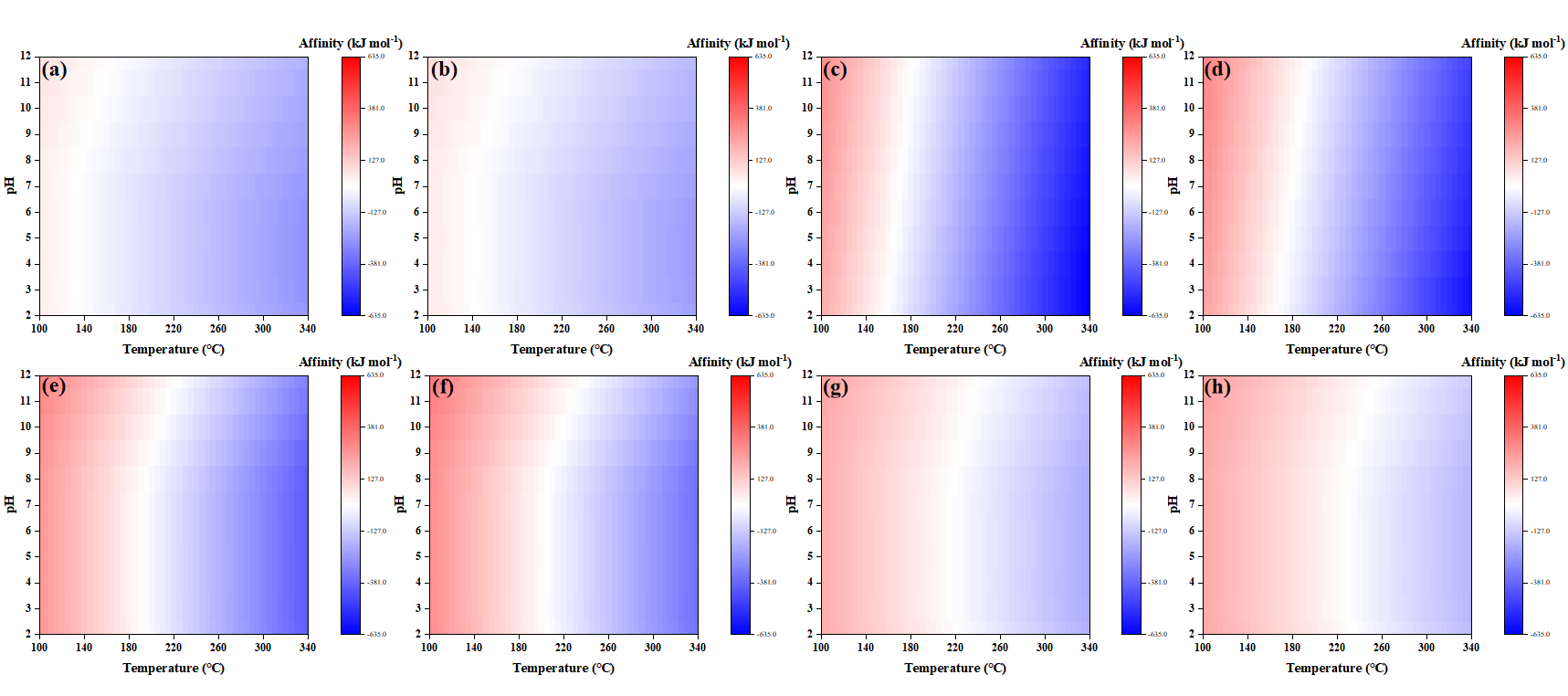


**Figure S6.** Synthesis affinity of threonine (a, b), tryptophan (c, d), tyrosine (e, f), and valine (g, h) using (a, c, e, g) CO,_g_ and (b, d, f, h) CO_2_,_g_ as the carbon source.

## Figures S7

**Figure S7.** Partial pressure of hydrogen gas constrained by the various minerals buffers (red: IW; blue: FQI; green: FMQ). Dashed line displays the value modeled by Zahnle et al. (2020) for the comparison.

## Supplementary Tables

**Table S1.** Some organic compounds and classifications in meteorites.

| **Types of organic matter** | **Representative substance** | **Chemical formula** | **References** |
| --- | --- | --- | --- |
| Amino Acids | glycine | C_2_H_5_NO_2_ | Kvenvolden et al., 1970;  Cronin and Pizzarello, 1983;  Cronin et al., 1982;  Pizzarello et al., 2004; |
|  | alanine | C_3_H_7_NO_2_ |  |
|  | proline | C_5_H_9_NO_2_ |  |
|  | leucine | C_6_H_13_NO_2_ |  |
|  | valine | C_5_H_11_NO_2_ |  |
|  | isoleucine | C_6_H_13_NO_2_ |  |
|  | glutamic acids | C_5_H_9_NO_4_ |  |
| Pyridine Carboxylic Acids | nicotinic acid | C_6_H_5_NO_2_ | Pizzarello et al., 2001 |
| Amines and Aliphatic Amides | sec-butyl amine | C_4_H_11_N | Pizzarello,. 2002b;  Pizzarello et al,. 1994 |
|  | Methylamine | CH_3_NH_2_ | Lawless J. G. et al.,1973  Jungclaus et al., 1976 |
|  | Ethylamine | C_2_H_7_N |  |
|  | Dimethylamine | C_2_H_7_N |  |
|  | n-Propylamine | C_3_H_9_N |  |
|  | Isopropylamine | C_3_H_9_N |  |
|  | n-Butylamine | C_4_H_11_N |  |
|  | Diethylamine | C_4_H_11_N |  |
|  | dicarboxylic acid monoamides | / | Cooper and Cronin, 1995;  Pizzarello and Huang, 2002 |
|  | hydroxy acid amides |  |  |
|  | lactams |  |  |
|  | carboxy lactams |  |  |
|  | lactims |  |  |
| Carboxylic Acids | Formic acid | CH_2_O_2_ | Yuen and Kvenvolden, 1974;  Lawless and Yuen, 1979;  Huang et al., 2005 |
|  | acetic acid | CH_3_COOH |  |
|  | Propionic acid | CH_3_CH_2_COOH |  |
|  | Isobutyric acid | C_4_H_8_O_2_ |  |
| Aliphatic Dicarboxylic Acids | Succinic | C_4_H_6_O_4_ | Pizzarello and Huang, 2002;  Shimoyama and Shigematsu, 1994 |
|  | Methylsuccinic | C_5_H_8_O_4_ |  |
|  | Glutaric | C_5_H_8_O_4_ |  |
|  | 3-methylglutaric | C_6_H_10_O_4_ |  |
|  | Adipic | C_6_H_10_O_4_ |  |
| Sulfonic and Phosphonic Acids | Methanesulfonic acid | CH_4_O_3_S | Cooper et al., 1992;  Cooper et al., 1997 |
|  | Ethanesulfonic acid | C_2_H_6_O_3_S |  |
|  | Methanephosphonic acid | CH_5_O_3_P |  |
| Alcohols and Carbonyls | 4C (R-OH/R-CHO)  5C (R-OH/R-CHO) | / | Jungclaus et al., 1976; |
| Hydroxy acids and Keto Acids | lactic acid | C_3_H_6_O_3_ | Peltzer and Bada, 1978;  Cooper et al., 2005 |
|  | α-hydroxy-n-butyric acid, | C_4_H_8_O_2_ |  |
|  | α-hy-droxy-n-valeric acid | C_5_H_10_O_2_ |  |
|  | acetoacetic acid | C_4_H_6_O_3_ |  |
| Polyhydroxylated organic compounds  (Polyols) | ethylene glycol | C_2_H_6_O_2_ | Cooper et al., 2001;  Crovisier et al., 2004;  Hollis et al., 2000, 2002;  Widicus-Weaver and Blake, 2005 |
|  | Glycerol | C_3_H_8_O_3_ |  |
|  | diadroxyacetone | C_3_H_6_O_3_ |  |
|  | Sugar alcohols | R-CHOH-CH_2_OH |  |
|  | sugar diacids | COOH-R-CHOH-COOH |  |
|  | deoxy-sugar  Acids | R-CHOH-CH_2_COOH |  |
| Aliphatic hydrocarbons | n-alkanes | C_n_H_2n+2_ | Pizzarello et al., 2001;  Kvenvolden et al., 1970 |
|  | isomeric alkanes |  |  |
|  | alkenes | C_n_H_2n_ |  |
| Aromatic hydrocarbons | Pyrene | C_16_H_10_ | Sephton, 2002;  Naraoka et al., 2000;  Sephton et al., 1998; |
|  | fluoranthene | C_16_H_10_ |  |
|  | phenanthrene | C_14_H_10_ |  |
|  | acenaphthene | C_12_H_8_ |  |
|  | naphthalene | C_10_H_8_ |  |
|  | biphenyl | C_12_H_10_ |  |
|  | anthracene | C_14_H_10_ |  |
| Polar Hydrocarbons | Nitrogen-containing heterocycles | / | Stoks and Schwartz, 1982;  Krishnamurthy et al., 1992;  Hayatsu et al., 1968; |
|  | - pyridines |  |  |
|  | - quinolines |  |  |
|  | - isoquinolines |  |  |
|  | - aromatic amides |  |  |
|  | Aromatic dicarboximides | / |  |
|  | - phthalimide |  |  |
|  | - homophthalimide |  |  |
| purine and pyrimidine nucleobases | Adenine | C_5_H_5_N_5_ | Stoks, P. G. 1979;  Yasuhiro Oba. et al., 2022 |
|  | Guanine | C_5_H_5_N_5_O |  |
|  | Isoguanine | C_5_H_5_N_5_O |  |
|  | Xanthine | C_5_H_4_N_4_O_2_ |  |

**Table S2.** Synthesis reactions involved in this study.

| **Chemvial** | **Reaction** |
| --- | --- |
| HCN, aq | CO_2_ + H_2_ = HCN + 2H_2_O |
|  | CO + NH_3_ = HCN + H_2_O |
|  | CH_4_ + NH_3_ = HCN + 3H_2_ |
| CN- | CO_2_ + H_2_ = CN^-^ + H^+^ + 2H_2_O |
|  | CO + NH_3_ = CN^-^ + H^+^ + H_2_O |
|  | CH_4_ + NH_3_ = CN^-^ + H^+^ + 3H_2_ |
| Formic-acid | CO_2_ + H_2_ = HCOOH |
|  | CO + H_2_O = HCOOH |
|  | CH_4_ + 2H_2_O = HCOOH + 3H_2_ |
| Formate | CO_2_ + H_2_ = COOH^-^ + H ^+^ |
|  | CO + H_2_O = COOH^-^ + H ^+^ |
|  | CH_4_ + 2H_2_O = COOH^-^ + H^+^ + 3H_2_ |
| CH4 | CO + 3H_2_ = CH_4_ + H_2_O |
|  | CO_2_ + 4H_2_ = CH_4_ + 2H_2_O |
| Formaldehyde | CO_2_ + 2H_2_ = HCHO + H_2_O |
|  | CO + H_2_ = HCHO |
|  | CH_4_ + H_2_O = HCHO + 2H_2_ |
| Methanol | CO_2_ + 3H_2_ = CH3OH + H_2_O |
|  | CO + 2H_2_ = CH_3_OH |
|  | CH_4_ + H_2_O = CH_3_OH + H_2_ |
| Acetic-acid | 2CO_2_ + 4H_2_ = C_2_H_4_O_2_ + 2H_2_O |
|  | 2CO + 2H_2_ = C_2_H_4_O_2_ |
|  | 2CH_4_ + 2H_2_O = C2H_4_O_2_ + 4H_2_ |
| Glycolic-acid | 2CO_2_+3H_2_=C_2_H_4_O_3_+H_2_O |
|  | 2CO+H_2_+H_2_O=C_2_H_4_O_3_ |
|  | 2CH_4_+3H_2_O=C2H_4_O_3_+5H_2_ |
| Propanoic-acid | 3CO_2_ + 7H_2_ = C3H_6_O_2_ + 4H_2_O |
|  | 3CO + 4H_2_ = C_3_H_6_O_2_ + H_2_O |
|  | 3CH_4_ + 2H_2_O = C_3_H_6_O_2_ + 5H_2_ |
| Pyruvic acid | 3CO_2_+5H_2_=C_3_H_4_O_3_+3H_2_O |
|  | 3CO+2H_2_=C_3_H_4_O_3_ |
|  | 3CH_4_+3H_2_O=C_3_H_4_O_3_+H_2_ |
| Valine | 5CO_2_ + NH_3_ + 12H_2_ = C_5_H_11_NO_2_ + 8H_2_O |
|  | 5CO + NH_3_ + 7H_2_ = C_5_H_11_NO_2_ + 3H_2_O |
|  | 5CH_4_ + NH_3_ + 2H_2_O = C_5_H_11_NO_2_ + 8H_2_ |
| Tyrosine | 9CO_2_ + NH_3_ + 19H_2_ = C_9_H_11_NO_3_ + 15H_2_O |
|  | 9CO + NH_3_ + 10H_2_ = C_9_H_11_NO_3_ + 6H_2_O |
|  | 9CH_4_ + NH_3_ + 3H_2_O = C_9_H^11^NO^3^ + 17H_2_ |
| Threonine | 4CO_2_ + NH_3_ + 8H_2_ = C_4_H_9_NO_3_ + 5H_2_O |
|  | 4CO + NH_3_ + 4H_2_ = C_4_H_9_NO_3_ + H_2_O |
|  | 4CH_4_ + NH_3_ + 3H_2_O = C_4_H_9_NO_3_ + 8H_2_ |
| Serine | 3CO_2_ + NH_3_ + 5H_2_ = C_3_H_7_NO_3_ + 3H_2_O |
|  | 3CO + NH_3_ + 2H_2_ = C_3_H_7_NO_3_ |
|  | 3CH_4_ + NH_3_ + 3H_2_O = C_3_H_7_NO_3_ + 7H_2_ |
| Proline | 5CO_2_ + NH_3_ + 11H_2_ = C_5_H_9_NO_2_ + 8H_2_O |
|  | 5CO + NH_3_ + 6H_2_ = C_5_H_9_NO_2_ + 3H_2_O |
|  | 5CH_4_ + NH_3_ + 2H_2_O = C_5_H_9_NO_2_ + 9H_2_ |
| Phenylalanine | 9CO_2_ + NH_3_ + 20H_2_ = C_9_H_11_NO_2_ + 16H_2_O |
|  | 9CO + NH_3_ + 11H_2_ = C_9_H_11_NO_2_ + 7H_2_O |
|  | 9CH_4_ + NH_3_ + 2H_2_O = C_9_H_11_NO_2_ + 16H_2_ |
| Lysine | 6CO_2_ + 2NH_3_ + 14H_2_ = C_6_H_14_N_2_O_2_ + 10H_2_O |
|  | 6CO + 2NH_3_ + 8H_2_ = C_6_H_14_N_2_O_2_ + 4H_2_O |
|  | 6CH_4_ + 2NH_3_ + 2H_2_O = C_6_H_14_N_2_O_2_ + 10H_2_ |
| Leucine | 6CO_2_ + NH_3_ + 15H_2_ = C_6_H_13_NO_2_ + 10H_2_O |
|  | 6CO + NH_3_ + 9H_2_ = C_6_H_13_NO_2_ + 4H_2_O |
|  | 6CH_4_ + NH_3_ + 2H_2_O = C_6_H_13_NO_2_ + 9H_2_ |
| Histidine | 6CO_2_ + 3NH_3_ + 10H_2_ = C_6_H_9_N_3_O_2_ + 10H_2_O |
|  | 6CO + 3NH_3_ + 4H_2_ = C_6_H_9_N_3_O_2_ + 4H_2_O |
|  | 6CH_4_ + 3NH_3_ + 2H_2_O = C_6_H_9_N_3_O_2_ + 14H_2_ |
| Glycine | 2CO_2_ + NH_3_ + 3H_2_ = C_2_H_5_NO_2_ + 2H_2_O |
|  | 2CO + NH_3_ + H_2_ = C_2_H_5_NO_2_ |
|  | 2CH_4_ + NH_3_ + 2H_2_O = C_2_H_5_NO_2_ + 5H_2_ |
| Glutamine | 5CO_2_ + 2NH_3_ + 9H_2_ = C_5_H_10_N_2_O_3_ + 7H_2_O |
|  | 5CO + 2NH_3_ + 4H_2_ = C_5_H_10_N_2_O_3_ + 2H_2_O |
|  | 5CH_4_ + 2NH_3_ + 3H_2_O = C_5_H_10_N_2_O_3_ + 11H_2_ |
| Asparticacid | 4CO_2_ + NH_3_ + 6H_2_ = C_4_H_7_NO_4_ + 4H_2_O |
|  | 4CO + NH_3_ + 2H_2_ = C_4_H_7_NO_4_ |
|  | 4CH_4_ + NH_3_ + 4H_2_O = C_4_H_7_NO_4_ + 10H_2_ |
| Arginine | 6CO_2_ + 4NH_3_ + 11H_2_ = C_6_H1_4_N_4_O_2_ + 10H_2_O |
|  | 6CO + 4NH_3_ + 5H_2_ = C_6_H14N4O2 + 4H_2_O |
|  | 6CH_4_ + 4NH_3_ + 2H_2_O = C_6_H_14_N_4_O_2_ + 13H_2_ |
| Alanine | 3CO_2_ + NH_3_ + 6H_2_ = C_3_H_7_NO_2_ + 4H_2_O |
|  | 3CO + NH_3_ + 3H_2_ = C_3_H_7_NO_2_ + H_2_O |
|  | 3CH_4_ + NH_3_ + 2H_2_O = C_3_H_7_NO_2_ + 6H_2_ |
| Glucose | 6CO_2_ + 12H_2_ = C_6_H_12_O_6_ + 6H_2_O |
|  | 6CO + 6H_2_ = C_6_H_12_O_6_ |
|  | 6CH_4_ + 6H_2_O = C_6_H_12_O_6_ + 12H_2_ |
| Ribose | 5CO_2_ + 10H_2_ = C_5_H_10_O_5_ + 5H_2_O |
|  | 5CO + 5H_2_ = C_5_H_10_O_5_ |
|  | 5CH_4_ + 5H_2_O = C_5_H_10_O_5_ + 10H_2_ |
| Deoxyribose | 5CO_2_ + 11H_2_ = C_5_H_10_O_4_ + 6H_2_O |
|  | 5CO + 6H_2_ = C_5_H_10_O_4_ + H_2_O |
|  | 5CH_4_ + 4H_2_O = C_5_H_10_O_4_ + 9H_2_ |
| Adenine | 5CO_2_ + 5NH_3_ + 5H_2_ = C_5_H_5_N_5_ + 10H_2_O |
|  | 5CO + 5NH_3_ = C_5_H_5_N_5_ + 5H_2_O |
|  | 5CH_4_ + 5NH_3_ = C_5_H_5_N_5_ + 15H_2_ |
| Cytosine | 4CO_2_ + 3NH_3_ + 5H_2_ = C_4_H_5_N_3_O + 7H_2_O |
|  | 4CO + 3NH_3_ + H_2_ = C_4_H_5_N_3_O + 3H_2_O |
|  | 4CH_4_ + 3NH_3_ + H_2_O = C_4_H_5_N_3_O + 11H_2_ |
| Guanine | 5CO_2_ + 5NH_3_ + 4H_2_ = C_5_H_5_N_5_O + 9H_2_O |
|  | 5CO + 5NH_3_ = C_5_H_5_N_5_O + 4H_2_O |
|  | 5CH_4_ + 5NH_3_ + H_2_O = C_5_H_5_N_5_O + 16H_2_ |
| Thymine | 5CO_2_ + 2NH_3_ + 8H_2_ = C_5_H_6_N_2_O_2_ + 8H_2_O |
|  | 5CO + 2NH_3_ + 3H_2_ = C_5_H_6_N_2_O_2_ + 3H_2_O |
|  | 5CH_4_ + 2NH_3_ + 2H_2_O = C_5_H_6_N_2_O_2_ + 12H_2_ |
| Uracil | 4CO_2_ + 2NH_3_ + 5H_2_ = C_4_H_4_N_2_O_2_ + 6H_2_O |
|  | 4CO + 2NH_3_ + H_2_ = C_4_H_4_N_2_O_2_ + 2H_2_O |
|  | 4CH_4_ + 2NH_3_ + 2H_2_O = C_4_H_4_N_2_O_2_ + 11H_2_ |
| Adenosine | 10CO_2_ + 5NH_3_ + 15H_2_ = C_10_H_13_N_5_O_4_ + 16H_2_O |
|  | 10CO + 5NH_3_ + 5H_2_ = C_10_H_13_N_5_O_4_ + 6H_2_O |
|  | 10CH_4_ + 5NH_3_ + 4H_2_O = C_10_H_13_N_5_O_4_ + 25H_2_ |
| Cytidine | 9CO_2_ + 3NH_3_ + 15H_2_ = C_9_H_13_N_3_O_5_ + 13H_2_O |
|  | 9CO + 3NH_3_ + 6H_2_ = C_9_H_13_N_3_O_5_ + 4H_2_O |
|  | 9CH_4_ + 3NH_3_ + 5H_2_O = C_9_H_13_N_3_O_5_ + 21H_2_ |
| Guanosine | 10CO_2_ + 5NH_3_ + 14H_2_ = C_10_H_13_N_5_O_5_ + 15H_2_O |
|  | 10CO + 5NH_3_ + 4H_2_ = C_10_H_13_N_5_O_5_ + 5H_2_O |
|  | 10CH_4_ + 5NH_3_ + 5H_2_O = C_10_H_13_N_5_O_5_ + 26H_2_ |
| Thymidine | 10CO_2_ + 2NH_3_ + 18H_2_ = C_10_H_14_N_2_O_6_ + 14H_2_O |
|  | 10CO + 2NH_3_ + 8H_2_ = C_10_H_14_N_2_O_6_ + 4H_2_O |
|  | 10CH_4_ + 2NH_3_ + 6H_2_O = C_10_H_14_N_2_O_6_ + 22H_2_ |
| Uridine | 9CO_2_ + 2NH_3_ + 15H_2_ = C_9_H_12_N_2_O_6_ + 12H_2_O |
|  | 9CO + 2NH_3_ + 6H_2_ = C_9_H_12_N_2_O_6_ + 3H_2_O |
|  | 9CH_4_ + 2NH_3_ + 6H_2_O = C_9_H_12_N_2_O_6_ + 21H_2_ |
| Deoxyadenosine | 10CO_2_ + 5NH_3_ + 16H_2_ = C_10_H_13_N_5_O_3_ + 17H_2_O |
|  | 10CO + 5NH_3_ + 6H_2_ = C_10_H_13_N_5_O_3_ + 7H_2_O |
|  | 10CH_4_ + 5NH_3_ + 3H_2_O = C_10_H_13_N_5_O_3_ + 24H_2_ |
| Deoxycytidine | 9CO_2_ + 3NH_3_ + 16H_2_ = C_9_H_13_N_3_O_4_ + 14H_2_O |
|  | 9CO + 3NH_3_ + 7H_2_ = C_9_H_13_N_3_O_4_ + 5H_2_O |
|  | 9CH_4_ + 3NH_3_ + 4H_2_O = C_9_H_13_N_3_O_4_ + 20H_2_ |
| Deoxyguanosine | 10CO_2_ + 5NH_3_ + 15H_2_ = C_10_H_13_N_5_O_4_ + 16H_2_O |
|  | 10CO + 5NH_3_ + 5H_2_ = C_10_H_13_N_5_O_4_ + 6H_2_O |
|  | 10CH_4_ + 5NH_3_ + 4H_2_O = C_10_H_13_N_5_O_4_ + 25H_2_ |
| Deoxythymidine | 10CO_2_ + 2NH_3_ + 19H_2_ = C_10_H_14_N_2_O_5_ + 15H_2_O |
|  | 10CO + 2NH_3_ + 9H_2_ = C_10_H_14_N_2_O_5_ + 5H_2_O |
|  | 10CH_4_ + 2NH_3_ + 5H_2_O = C_10_H_14_N_2_O_5_ + 21H_2_ |
| Deoxyuridine | 9CO_2_ + 2NH_3_ + 16H_2_ = C_9_H_12_N_2_O_5_ + 13H_2_O |
|  | 9CO + 2NH_3_ + 7H_2_ = C_9_H_12_N_2_O_5_ + 4H_2_O |
|  | 9CH_4_ + 2NH_3_ + 5H_2_O = C_9_H_12_N_2_O_5_ + 20H_2_ |
| Urea | CO_2_ + 2NH_3_ = CH_4_N_2_O + H_2_O |
|  | CO + 2NH_3_ = CH_4_N_2_O + H_2_ |
|  | CH_4_ + 2NH_3_ + H_2_O = CH_4_N_2_O + 4H_2_ |

**Table S3.** Thermodynamic properties (standard molar Gibbs free energy, enthalpy, entropy, heat capacity, volume, and Helgeson-Kirkham- Flowers parameters) of the organics considered in this study.

| Chemical | ΔG_f_^0^ | ΔH_f_^0^ | S^0^ | V^0^ | C_p_^0^ | ω x 10^-5^ | a_1_ x 10 | a_2_ x 10^-2^ | a_3_ | a_4_ x 10^-4^ | c_1_ | c_2_ x 10^-4^ | References |
| --- | --- | --- | --- | --- | --- | --- | --- | --- | --- | --- | --- | --- | --- |
| Name | cal mol^-1^ | cal mol^-1^ | cal mol^-1^ K^-1^ | cm^3^ mol^-1^ | cal mol^-1^ K^-1^ | cal mol^-1^ | cal mo^l-1^ bar^-1^ | cal mol^-1^ | cal K mol^-1^ bar^-1^ | cal K mol^-1^ | cal mol^-1^ K^-1^ | cal K mol^-1^ |  |
| Formate | -83862 | -101680 | 21.70 | 26.16 | -22.00 | 1.30 | 5.27 | 7.68 | 3.47 | -3.10 | 17.00 | -12.40 | (Kitadai, 2015) |
| Formic Acid | -88982 | -101680 | 38.90 | 34.69 | 19.02 | -0.34 | 8.09 | 3.42 | 1.97 | -2.92 | 22.19 | -3.12 |  |
| Methanol | -42050 | -58870 | 31.60 | 38.17 | 37.80 | -0.15 | 8.40 | 4.10 | -0.24 | -1.43 | 34.40 | 0.96 |  |
| Acetate | -88270 | -116180 | 20.60 | 40.50 | 6.31 | 1.32 | 10.02 | 2.81 | 6.72 | -2.89 | 26.30 | -3.86 |  |
| Acetic Acid | -94760 | -116100 | 42.70 | 52.01 | 41.08 | -0.08 | 11.50 | 5.50 | 1.67 | -2.87 | 44.90 | -2.63 |  |
| Propanoate | -86770 | -122630 | 26.50 | 54.95 | 32.46 | 1.23 | 8.38 | 19.34 | 0.05 | -3.58 | 52.30 | -4.20 |  |
| Propanoic Acid | -93450 | -122470 | 49.40 | 68.00 | 60.90 | -0.09 | 14.60 | 7.90 | 6.90 | -4.30 | 63.30 | -1.40 |  |
| Hydrocyanic acid | 28600 | 26500 | 29.80 | 45.90 | 40.70 | -0.11 | 8.01 | 11.77 | 1.13 | -3.27 | 28.99 | 5.26 |  |
| Cyanide | 41200 | 36000 | 22.50 | 24.20 | -32.70 | 1.29 | 5.51 | 5.68 | 3.52 | -3.01 | -1.11 | -9.70 |  |
| Alanine | -88810 | -132500 | 38.83 | 60.40 | 33.60 | 0.18 | 14.90 | 1.74 | 7.16 | -3.69 | 49.50 | -7.00 |  |
| Alanine+ | -92000 | -133200 | 47.18 | 67.90 | 64.50 | -0.21 | 20.02 | -6.38 | -7.07 | -0.88 | 61.60 | 0.46 |  |
| Alanine− | -75360 | -121590 | 30.30 | 59.50 | 18.00 | -0.32 | 12.55 | 4.99 | 18.91 | -5.78 | 28.90 | -5.87 |  |
| Arginine | -57360 | -143060 | 80.06 | 123.90 | 67.40 | 0.22 | 28.83 | 8.21 | 7.20 | -5.95 | 94.80 | -12.50 |  |
| Arginine+ | -69630 | -153860 | 84.98 | 122.50 | 77.60 | 0.73 | 31.72 | 0.22 | 5.46 | -5.15 | 103.80 | -9.60 |  |
| Arginine− | -41070 | -142650 | 26.82 | 143.00 | 81.10 | 2.30 | 31.28 | 13.76 | 32.12 | -9.94 | 141.90 | -19.60 |  |
| Arginine2+ | -72110 | -154860 | 89.96 | 132.60 | 105.50 | 0.90 | 38.92 | -8.28 | -15.70 | -2.21 | 120.30 | -3.25 |  |
| Asparagine | -125490 | -182700 | 57.88 | 77.40 | 30.80 | 0.21 | 19.83 | 2.37 | 3.76 | -4.81 | 56.50 | -11.70 |  |
| Asparagine+ | -128440 | -183630 | 64.65 | 85.70 | 60.70 | 0.81 | 24.64 | -4.50 | -4.58 | -2.24 | 68.00 | 0.06 |  |
| Asparagine− | -113250 | -172960 | 49.51 | 77.10 | 16.50 | 2.10 | 17.80 | 6.85 | 23.16 | -7.12 | 44.60 | -4.36 |  |
| Asparticacid | -172510 | -226340 | 55.22 | 74.70 | 32.40 | 0.17 | 18.96 | 3.05 | -6.62 | -2.23 | 58.10 | -11.90 |  |
| Asparticacid+ | -175170 | -227970 | 58.68 | 84.90 | 58.10 | -0.73 | 22.74 | -4.58 | 0.19 | -2.18 | 58.90 | -3.66 |  |
| Asparticacid− | -167380 | -225180 | 41.90 | 64.80 | 2.70 | 2.47 | 16.96 | 5.77 | 10.11 | -6.37 | 56.30 | -15.30 |  |
| Asparticacid^2−^ | -153520 | -215330 | 28.43 | 64.80 | -9.90 | 3.16 | 15.19 | 9.10 | 23.54 | -8.14 | 44.40 | -12.45 |  |
| Glutamine | -126280 | -191860 | 62.41 | 93.70 | 43.80 | 0.18 | 23.22 | 3.58 | 5.44 | -5.23 | 68.60 | -11.40 |  |
| Glutamine+ | -129240 | -192540 | 70.05 | 102.70 | 73.10 | 0.90 | 28.42 | -2.72 | -2.17 | -3.52 | 81.40 | 0.02 |  |
| Glutamine− | -113720 | -182180 | 52.75 | 93.10 | 28.70 | 2.19 | 21.44 | 8.52 | 24.95 | -8.33 | 58.00 | -4.48 |  |
| Glutamicacid | -173050 | -234820 | 61.20 | 90.20 | 43.50 | 0.15 | 22.30 | 4.23 | 6.55 | -5.84 | 65.20 | -10.00 |  |
| Glutamicacid+ | -176050 | -235700 | 68.31 | 98.10 | 65.70 | -2.20 | 25.66 | -3.20 | -5.05 | -3.17 | 54.40 | -4.36 |  |
| Glutamicacid− | -167250 | -233860 | 44.97 | 80.00 | 7.60 | 2.65 | 20.49 | 6.96 | 10.86 | -7.02 | 47.60 | -7.80 |  |
| Glutamicacid^2−^ | -153650 | -224900 | 29.42 | 79.70 | -5.30 | 3.24 | 19.02 | 10.67 | 22.11 | -9.27 | 34.80 | -5.11 |  |
| Glycine | -88620 | -122830 | 37.89 | 43.20 | 9.30 | 0.23 | 11.30 | 0.71 | 3.99 | -3.04 | 28.50 | -8.40 |  |
| Glycine+ | -91820 | -123790 | 45.44 | 49.90 | 39.70 | -0.33 | 15.57 | -8.26 | -6.62 | 0.47 | 39.50 | -1.21 |  |
| Glycine− | -75270 | -112270 | 28.57 | 45.70 | -0.80 | 0.69 | 9.76 | 3.54 | 19.85 | -4.73 | 21.60 | -7.92 |  |
| Histidine | -48420 | -108200 | 66.00 | 99.30 | 56.20 | 0.27 | 24.32 | 4.80 | 7.52 | -6.05 | 81.60 | -11.30 |  |
| Histidine+ | -56570 | -115300 | 69.50 | 97.00 | 59.10 | 0.50 | 26.64 | -3.40 | -0.88 | -3.07 | 88.00 | -12.00 |  |
| Histidine− | -35760 | -97700 | 58.75 | 97.70 | 37.90 | 0.62 | 21.36 | 9.00 | 25.83 | -8.67 | 61.90 | -7.79 |  |
| Histidine^2+^ | -58750 | -116300 | 73.47 | 106.10 | 87.80 | 0.69 | 32.48 | -11.06 | -16.13 | -0.21 | 102.90 | -4.28 |  |
| Isoleucine | -81990 | -151600 | 49.70 | 105.80 | 91.60 | 0.09 | 24.49 | 6.55 | 18.16 | -7.76 | 99.70 | -3.60 |  |
| Isoleucine+ | -85190 | -151830 | 59.64 | 115.10 | 120.50 | 0.39 | 31.68 | -1.42 | -6.57 | -4.46 | 121.00 | 1.49 |  |
| Isoleucine− | -68750 | -140690 | 41.92 | 106.10 | 77.60 | 0.75 | 24.15 | 9.89 | 21.15 | -9.31 | 96.00 | -4.90 |  |
| Leucine | -84200 | -153600 | 50.41 | 107.80 | 95.20 | 0.09 | 24.68 | 7.51 | 19.93 | -8.37 | 102.70 | -3.30 |  |
| Leucine+ | -87380 | -153990 | 59.75 | 117.30 | 122.40 | 1.22 | 31.28 | -1.18 | 3.94 | -4.63 | 119.10 | 7.09 |  |
| Leucine− | -70910 | -142750 | 42.22 | 106.90 | 77.00 | 2.51 | 24.18 | 9.97 | 30.41 | -9.37 | 95.50 | 2.20 |  |
| Lysine | -80680 | -159330 | 57.91 | 108.60 | 63.80 | 0.07 | 24.56 | 8.30 | 24.43 | -9.53 | 86.60 | -10.90 |  |
| Lysine+ | -93080 | -168720 | 68.00 | 107.50 | 67.90 | 1.21 | 28.62 | -1.51 | 4.47 | -3.99 | 95.20 | -8.00 |  |
| Lysine− | -65810 | -148950 | 42.87 | 112.20 | 72.80 | 2.67 | 28.29 | 10.53 | 2.97 | -7.61 | 133.70 | -18.00 |  |
| Lysine^2+^ | -95610 | -168800 | 76.20 | 117.00 | 96.20 | 1.41 | 35.18 | -9.91 | -13.36 | -1.03 | 110.80 | -0.83 |  |
| Phenylalanine | -49430 | -108900 | 56.60 | 122.10 | 93.80 | 0.12 | 28.27 | 8.80 | 19.34 | -9.38 | 108.10 | -6.50 |  |
| Phenylalanine+ | -52430 | -109500 | 64.65 | 132.20 | 121.00 | 1.13 | 35.42 | 0.38 | -0.44 | -5.75 | 125.10 | 3.12 |  |
| Phenylalanine− | -36730 | -98200 | 49.89 | 120.90 | 75.70 | 2.42 | 28.15 | 11.44 | 25.73 | -10.43 | 101.50 | -1.74 |  |
| Proline | -73560 | -124100 | 49.83 | 82.60 | 43.00 | 0.14 | 19.39 | 4.87 | 11.88 | -5.68 | 63.60 | -9.50 |  |
| Proline+ | -76220 | -124440 | 57.61 | 88.50 | 73.00 | -0.77 | 24.19 | -4.21 | -4.18 | -2.45 | 70.10 | -2.05 |  |
| Proline− | -59040 | -113790 | 35.72 | 82.90 | 40.80 | 0.70 | 16.94 | 7.45 | 32.30 | -7.55 | 59.90 | -5.41 |  |
| Serine | -123930 | -172420 | 47.24 | 60.60 | 27.80 | 0.18 | 15.69 | 0.73 | 3.87 | -3.49 | 48.50 | -9.40 |  |
| Serine+ | -126910 | -173740 | 52.82 | 68.50 | 58.70 | -0.82 | 19.13 | -6.30 | -2.53 | -0.94 | 56.30 | -2.52 |  |
| Serine− | -111370 | -162070 | 39.82 | 60.40 | 12.30 | 0.35 | 13.15 | 5.09 | 19.39 | -5.85 | 37.10 | -9.20 |  |
| Threonine | -119830 | -178940 | 44.23 | 77.60 | 50.10 | 0.11 | 18.94 | 2.83 | 8.87 | -4.87 | 65.50 | -7.10 |  |
| Threonine+ | -122910 | -179820 | 51.61 | 86.10 | 78.40 | -0.48 | 23.15 | -4.46 | 0.49 | -2.27 | 77.20 | -1.57 |  |
| Threonine− | -107330 | -169020 | 35.59 | 75.80 | 34.70 | -0.22 | 15.31 | 6.71 | 27.60 | -7.02 | 46.90 | -6.02 |  |
| tyrosine | -91800 | -157740 | 59.41 | 124.50 | 80.20 | 0.09 | 30.06 | 8.49 | 8.36 | -8.58 | 106.20 | -12.40 |  |
| tyrosine+ | -94580 | -157400 | 69.88 | 134.70 | 108.00 | 1.22 | 37.25 | 0.64 | -8.74 | -5.94 | 123.00 | -1.88 |  |
| tyrosine− | -79400 | -148090 | 50.19 | 111.80 | 51.20 | 2.78 | 26.34 | 10.49 | 16.42 | -7.59 | 109.00 | -16.00 |  |
| tyrosine^2−^ | -64820 | -136740 | 39.35 | 101.60 | 15.30 | 3.48 | 24.63 | 12.97 | 20.83 | -10.93 | 91.40 | -21.73 |  |
| Valine | -85330 | -146420 | 45.68 | 90.90 | 74.00 | 0.12 | 21.35 | 4.48 | 14.58 | -6.12 | 83.80 | -4.30 |  |
| Valine+ | -88450 | -146590 | 55.57 | 100.40 | 103.50 | 0.08 | 27.20 | -2.96 | -1.29 | -3.35 | 102.50 | 0.87 |  |
| Valine− | -72070 | -138360 | 28.23 | 88.80 | 54.80 | 0.79 | 20.38 | 8.07 | 18.60 | -8.00 | 76.80 | -6.27 |  |
| Adenine | 74770 | 31235 | 53.41 | 89.59 | 56.24 | 0.07 | 21.50 | 8.50 | -2.66 | -5.36 | 87.88 | -15.87 | (LaRowe and Helgeson, 2006) |
| Guanine | 16702 | -34803 | 51.19 | 97.90 | 60.06 | 0.23 | 23.30 | 7.46 | -5.48 | -3.41 | 90.14 | -15.87 |  |
| Cytosine | -8411 | -46915 | 47.64 | 73.35 | 41.81 | 0.17 | 17.31 | 5.86 | 6.60 | -5.22 | 70.51 | -13.36 |  |
| Thymine | -61315 | -104969 | 48.96 | 88.04 | 56.93 | 0.12 | 21.27 | 7.27 | -1.03 | -5.09 | 83.20 | -12.39 |  |
| Uracil | -59734 | -94587 | 45.88 | 71.55 | 36.19 | 0.14 | 17.50 | 5.93 | -1.61 | -4.31 | 56.57 | -9.40 |  |
| Ribose | -179739 | -247132 | 59.53 | 95.71 | 66.46 | 0.17 | 22.65 | 7.29 | -5.39 | -3.41 | 134.70 | -32.82 |  |
| Deoxyribose | -144433 | -205037 | 57.82 | 94.65 | 56.16 | 0.17 | 22.54 | 7.27 | -5.37 | -3.40 | 124.40 | -32.82 |  |
| Adenosine | -46499 | -148490 | 87.19 | 170.70 | 120.30 | 0.23 | 39.55 | 12.90 | 8.97 | -8.82 | 163.20 | -20.10 |  |
| Cytidine | -130179 | -226640 | 83.09 | 153.30 | 96.38 | 0.34 | 36.31 | 8.00 | 7.59 | -6.59 | 132.60 | -16.30 |  |
| Guanosine | -105568 | -214528 | 88.33 | 174.20 | 124.90 | 0.39 | 42.26 | 8.38 | -2.46 | -5.89 | 169.30 | -20.10 |  |
| Thymidine | -183083 | -284694 | 84.42 | 167.40 | 110.50 | 0.29 | 38.16 | 10.96 | 12.40 | -7.19 | 143.40 | -14.90 |  |
| Uridine | -181502 | -274312 | 81.34 | 151.80 | 95.14 | 0.31 | 36.15 | 7.09 | 5.13 | -5.68 | 131.80 | -16.65 |  |
| Deoxyadenosine | -11193 | -106386 | 85.48 | 170.00 | 111.20 | 0.23 | 38.03 | 16.96 | 15.16 | -10.83 | 154.20 | -20.10 |  |
| Deoxycytidine | -96652 | -184534 | 87.35 | 154.00 | 87.28 | 0.34 | 35.82 | 9.72 | 7.40 | -6.53 | 123.50 | -16.30 |  |
| Deoxyguanosine | -71539 | -176273 | 77.99 | 173.20 | 115.80 | 0.39 | 39.45 | 15.50 | 14.28 | -10.57 | 160.30 | -20.10 |  |
| Deoxythymidine | -149556 | -242588 | 88.68 | 167.50 | 101.40 | 0.29 | 38.87 | 11.45 | -7.78 | -3.61 | 134.40 | -14.90 |  |
| Deoxyuridine | -147975 | -232206 | 85.59 | 151.20 | 86.04 | 0.31 | 35.59 | 9.62 | 3.50 | -6.28 | 122.80 | -16.65 |  |
| Formaldehyde | -26131 | -33890 | 28.50 | 26.90 | 15.70 | -0.40 | 5.31 | 5.31 | 3.39 | -3.00 | 18.96 | -3.41 | (Schulte & Shock, 1993) |
| Pyruvic acid | -117000 | -140300 | 62.1 | 64.6 | 36.3 | -0.176 | 10.5381 | 17.1584 | 0.7032 | -3.4883 | 37.4903 | -1.3746 | (Peter A. Canovas, 2016) |
| Pyruvate- | -113600 | -137400 | 60.4 | 51.5 | -3.7 | 0.7144 | 9.0836 | 13.862 | 1.4526 | -3.3521 | 11.2428 | -4.1508 |  |
| Glycolic Acid | -126400 | -154890 | 43.10 | 51.80 | 45.50 | -0.30 | 8.74 | 12.15 | 4.02 | -3.28 | 43.67 | -0.47 |  |
| Glycolate | -121170 | -154700 | 26.20 | 39.50 | 6.60 | 1.23 | 7.63 | 11.06 | 0.98 | -3.24 | 26.05 | -4.03 |  |
| Glucose | -218905 | -301671 | 64.89 | 112.20 | 86.76 | -1.58 | 25.53 | 3.11 | 12.38 | -4.88 | 89.72 | -7.89 | (Amend & Plyasunov, 2001) |
| Tryptophan | -26.82 | -97.59 | 59.96 | 99.60 | 143.60 | 0.15 | 35.91 | 9.21 | 14.78 | -9.36 | 116.80 | -7.80 | (Kitadai, 2015) |
| Tryptophan+ | -30.05 | -97.52 | 71.04 | 126.60 | 154.50 | 1.02 | 41.24 | 2.72 | -4.16 | -7.44 | 134.70 | 0.62 |  |
| Tryptophan- | -14.05 | -86.88 | 42.99 | 81.10 | 141.90 | 2.31 | 33.75 | 13.65 | 21.44 | -12.02 | 111.00 | -4.28 |  |

**References**

Cronin J. R., and Pizzarello S. (1983). Amino acids in meteorites. Adv. Space Res. 3, 5–18. doi: 10.1016/0273-1177(83)90036-4

Cronin J. R., Yuen G. U., and Pizzarello S. (1982). Gas chromatographic-mass spectral analyses of the five-carbon β-, γ-, and δ-amino alkanoic acids. Anal. Biochem. 124, 139–149. doi: 10.1016/0003-2697(82)90231-7

Cooper G. W., and Cronin J. R. (1995). Linear and cyclic aliphatic carboxamides of the Murchison meteorite: Hydrolyzable derivatives of amino acids and other carboxylic acids. Geochim. Cosmochim. Acta. 59, 1003–1015. doi: 10.1016/0016-7037(95)00018-6

Cooper G. W., Onwo W. M., and Cronin J. R. (1992). Alkyl phosphonic acids and sulfonic acids in the Murchison meteorite. Geochim. Cosmochim. Acta. 56, 4109–4115. doi: 10.1016/0016-7037(92)90023-C

Cooper G. W., Thiemens M. H., Jackson T., and Chang S. (1997). Sulfur and hydrogen isotope anomalies in meteoritic sulfonic acids. Science. 277, 1072–1074. [doi: 10.1126/science.277.5329.1072](https://doi.org/10.1126/science.277.5329.1072)

Cooper G., Dugas A., Byrd A., Chang P. M., and Washington N. (2005). Keto-acids in carbonaceous meteorites (abstract). In Lunar and Planetary Science XXXVI, Abstract #2381. Lunar and Planetary Institute, Houston (CD-ROM).

Cooper G., Kimmich N., Belisle W., Sarinana J., Brabham K., and Garrel L. (2001). Carbonaceous meteorites as a source of sugarrelated organic compounds for the early Earth. Nature. 414, 879–883. doi: 10.1038/414879a

Crovisier J., Bockelée-Morvan D., Biver N., Colom P., Despois D., and Lis D. C. (2004). Ethylene glycol in comet C/1995 O1 (Hale-Bopp). Astron. Astrophys. 418, L35–L38. doi: 10.1051/0004-6361:20040116

Daniel Kopetzki and Markus Antonietti.(2011). Hydrothermal formose reaction. New J. Chem. 35, 1787–1794. doi: 10.1039/c1nj20191c

Huang Y., Wang Y., Alexandre M. R, Lee T., Rose-Petruck C., Fuller M., and Pizzarello S. (2005). Molecular and compound specific isotopic characterization of monocarboxylic acids in carbonaceous meteorites. Geochim. Cosmochim. Acta. 69, 1073–1084. doi: 10.1016/j.gca.2004.07.030

Hollis J. M., Lovas F. J., and Jewell P. R. (2000). Interstellar glycolaldehyde: The first sugar. Astrophys. J. Lett. 540, L107– L110. doi: 10.1086/312881

Hollis J. M., Lovas F. J., Jewell P. R., and Coudert L. H. (2002). Interstellar antifreeze: Ethylene glycol. Astrophys. J. Lett. 571, L59–L62. doi: 10.1086/341148

Hayatsu R., Studier M. H., Oda A., Fuse K., Anders E. (1968). Origin of organic matter in the early solar system-II. Nitrogen compounds. Geochim. Cosmochim. Acta. 32, 175–190. doi: 10.1016/S0016-7037(68)80003-1

Jungclaus G., Cronin J. R., Moore C. B., and Yuen G. U. (1976). Aliphatic amines in the Murchison meteorite. Nature. 261, 126–128. doi: 10.1038/261126a0

Kvenvolden K., Lawless J., Pering K., Peterson E., Flores J., Ponnamperuma C., Kaplan J. R., and Moore C. (1970). Evidence of extraterrestrial amino acids and hydrocarbons in the Murchison meteorite. Nature. 228, 923–926. doi: 10.1038/228923a0

Kvenvolden K., Lawless J., Pering K., Peterson E., Flores J., Ponnamperuma C., Kaplan J. R., and Moore C. (1970). Evidence of extraterrestrial amino acids and hydrocarbons in the Murchison meteorite. Nature. 228, 923–926. doi: 10.1038/228923a0

Krishnamurthy R. V., Epstein S., Cronin J. R., Pizzarello S., and Yuen G. U. (1992). Isotopic and molecular analyses of hydrocarbons and monocarboxylic acids in the Murchison meteorite. Geochim. Cosmochim. Acta. 56, 4045–4058. doi: 10.1016/0016-7037(92)90015-B

Lawless [J. G. (1973). Amino acids in the Murchison meteorite. Geochim. Cosmochim. Acta. 37, 2207-2212. doi:](https://www.sciencedirect.com/science/article/abs/pii/0016703773900173#!) [[10.1016/0016-7037(73)90017-3](https://www.sciencedirect.com/science/article/abs/pii/0016703773900173#!)](https://doi.org/10.1016/0016-7037(73)90017-3)

Lawless J. G., and Yuen G. (1979). Quantification of monocarboxylic acids in the Murchison carbonaceous meteorite. Nature. 282, 396–398. doi: 10.1038/282396a0

Matthew Levy and Stanley L. Miller. (1998). The stability of the RNA bases: Implications for the origin of life. Proc. Natl. Acad. Sci. USA. Vol 95, 7933-7938. doi: 10.2307/45680

Naraoka H., Shimoyama A., and Harada K. (2000). Isotopic evidence from an Antarctica carbonaceous chondrite for two reaction pathways of extraterrestrial PAH formation. Earth Planet. Sci. Lett. 184, 1–7. doi: 10.1016/S0012-821X(00)00316-2

Peter G. Stoks, Alan W. Schwartz. (1979). [Uracil in carbonaceous meteorites](https://www.x-mol.com/paperRedirect/1406763932378935296). Nature. 282, 709-710. doi: [10.1038/282709a0](https://www.x-mol.com/paperRedirect/1406763932378935296)

Pizzarello S. and Weber A. L. (2004). Prebiotic amino acids as asymmetric catalysts. Science. 303, 1151. doi: 10.2307/3836285

Pizzarello S., and Cooper G. W. (2001). Molecular and chiral analyses of some protein amino acid derivatives in the Murchison and Murray meteorites. Meteoritics & Planet. Sci. 36, 897– 909. doi: 10.1111/j.1945-5100.2001.tb01929.x

Pizzarello S. (2002b). The chiral amines of the Murchison meteorite: A preliminary characterization (abstract). In Lunar and Planetary Science XXXIII, Abstract #1233. Lunar and Planetary Institute, Houston (CD-ROM).

Pizzarello S., Feng X., Epstein S., and Cronin J. R. (1994). Isotopic analyses of nitrogenous compounds from the Murchison meteorite: Ammonia, amines, amino acids, and polar hydrocarbons. Geochim. Cosmochim. Acta. 58, 5579–5587. doi: 10.1016/0016-7037(94)90251-8

Pizzarello S., and Huang Y. (2002). Molecular and isotopic analyses of Tagish Lake alkyl dicarboxylic acids. Meteoritics & Planet. Sci. 37, 687–696. doi: [10.1111/j.1945-5100.2002.tb00848.x](https://doi.org/10.1111/j.1945-5100.2002.tb00848.x)

Peltzer E. T., and Bada J. L. (1978). α-hydroxycarboxylic acids in the Murchison meteorite. Nature. 272, 443–444. doi: 10.1038/272443a0

Robert A. Sanchez., James P. Ferbis., and Leslie E. Orgel. (1967). Studies in Prebiodc Synthesis Ⅱ Synthesis of Purine Precursors and Amino Acids from Aqueous Hydrogen Cyanide. J. Mol. Biol. 80, 223-253. doi:
10.1016/s0022-2836(67)80037-8

Shimoyama A., and Shigematsu R. (1994). Dicarboxylic-acids in the Murchison and Yamato-791198 carbonaceous chondrites. Chem. Lett. 3, 523–526. doi: 10.1246/cl.1994.523

Sephton M. A. (2002). Organic compounds in carbonaceous meteorites. Natl. Prod. Rep. 19, 292–311. doi: 10.1039/B103775G

Sephton M. A., Pillinger C. T., and Gilmour I. (1998). δ13C of free and macromolecular aromatic structures in the Murchison meteorite. Geochim. Cosmochim. Acta. 62, 1821–1828. doi: 10.1016/S0016-7037(98)00108-2

Stoks P. G., and Schwartz A. W. (1982). Basic nitrogen heterocyclic compounds in the Murchison meteorite. Geochim. Cosmochim. Acta. 46, 309–315. doi: 10.1016/0016-7037(82)90222-8

Shin Miyakawa , H. James Cleaves, Stanley L. Miller. (2002). [The cold origin of life: A. Implications based on the hydrolytic stabilities of hydrogen cyanide and formamide.](https://www.x-mol.com/paperRedirect/1213060951355625472) [Origins of Life and Evolution of Biospheres](https://www.x-mol.com/paper/journal/1907?r_detail=1213060951355625472). 32, 195-208. doi: 10.1023/A:1016514305984

Takashi Moriyoshi , Keisuke Sam and Yasuhiro Uosaki. (2001). Hydrothermal decomposition of esters under high

Pressure. [High Pressure Research. 20(1-6), 491-505.](https://wwwtandfonline.53yu.com/journals/ghpr20)doi: 10.1080/08957950108206197

Widicus-Weaver S. L., and Blake G. A. (2005). 1,3-Dihydroxyacetone in Sgr B2(N-LMH): The first interstellar ketose. Astrophys. J. Lett. 624, L33–L36. doi: 10.1086/497963

[Yasuhiro Oba](https://www.nature.com/articles/s41467-022-29612-x#auth-Yasuhiro-Oba), [Yoshinori Takano](https://www.nature.com/articles/s41467-022-29612-x#auth-Yoshinori-Takano), [Yoshihiro Furukawa](https://www.nature.com/articles/s41467-022-29612-x#auth-Yoshihiro-Furukawa), [Toshiki Koga](https://www.nature.com/articles/s41467-022-29612-x#auth-Toshiki-Koga), [Daniel P. Glavin](https://www.nature.com/articles/s41467-022-29612-x#auth-Daniel_P_-Glavin), [Jason P. Dworkin](https://www.nature.com/articles/s41467-022-29612-x#auth-Jason_P_-Dworkin) and [Hiroshi Naraoka](https://www.nature.com/articles/s41467-022-29612-x#auth-Hiroshi-Naraoka). (2022). Identifying the wide diversity of extraterrestrial purine and pyrimidine nucleobases in carbonaceous meteorites. Nature Communications. Online

Yuen G. U., and Kvenvolden K. A. (1974). Monocarboxylic acids in Murray and Murchison carbonaceous meteorites. Nature. 246, 301–303. doi: 10.1038/246301a0
